# Genes that are Used Together are More Likely to be Fused Together in Evolution by Mutational Mechanisms: A Bioinformatic Test of the Used-Fused Hypothesis

**DOI:** 10.1007/s11692-022-09579-9

**Published:** 2022-11-30

**Authors:** Evgeni Bolotin, Daniel Melamed, Adi Livnat

**Affiliations:** 1 Department of Evolutionary and Environmental Biology, University of Haifa, 3498838 Haifa, Israel; 2 Institute of Evolution, University of Haifa, Haifa, 3498838 Israel

**Keywords:** Fusion mutation, Translocation mutation, Exon shuffling, Genome organization, Parallelism, Nonrandom mutation

## Abstract

Cases of parallel or recurrent gene fusions in evolution as well as in genetic disease and cancer are difficult to explain, because unlike point mutations, they can require the repetition of a similar configuration of multiple breakpoints rather than the repetition of a single point mutation. The used-together-fused-together hypothesis holds that genes that are used together repeatedly and persistently in a specific context are more likely to undergo fusion mutation in the course of evolution for mechanistic reasons. This hypothesis offers to explain gene fusion in both evolution and disease under one umbrella. Using bioinformatic data, we tested this hypothesis against alternatives, including that all gene pairs can fuse by random mutation, but among pairs thus fused, those that had interacted previously are more likely to be favored by selection. Results show that across multiple measures of gene interaction, human genes whose orthologs are fused in one or more species are more likely to interact with each other than random pairs of genes of the same genomic distance between pair members; that an overlap exists between genes that fused in the course of evolution in non-human species and genes that undergo fusion in human cancers; and that across six primate species studied, fusions predominate over fissions and exhibit substantial evolutionary parallelism. Together, these results support the used-together-fused-together hypothesis over its alternatives. Multiple implications are discussed, including the relevance of mutational mechanisms to the evolution of genome organization, to the distribution of fitness effects of mutation, to evolutionary parallelism and more.

**Supplementary Information:**

The online version contains supplementary material available at 10.1007/s11692-022-09579-9.

## Introduction

TRIM5 is a restriction factor that recognizes and inactivates retroviral capsids (Virgen et al., 2008). CypA is a highly abundant cytosolic protein that, among its other roles, potently binds several retroviral capsids, including HIV-1 (Haendler & Hofer, 1990; Kaessmann et al., 2009). The genes encoding these proteins fused at least twice independently through retroposition in different simian lineages (Virgen et al., 2008; Nisole et al., 2004; Sayah et al., 2004; Liao et al., 2007; Brennan et al., 2008; Wilson et al., 2008; Newman et al., 2008), producing a fused gene that provides resistance to certain lentiviruses (Nisole et al., 2004; Sayah et al., 2004). It is hard to explain the independent origination of such a fusion by chance, because here, multiple similar breakpoints defining the same two loci have to appear independently by chance twice. If the probability of the independent origination of a given point mutation is small, the probability of the independent origination of multiple breakpoints is negligible. Moreover, multiple other *TRIM* genes exist, and in vitro studies have shown that fusions of *CypA* to them would have also provided some, though smaller, resistance to retroviruses (Zhang et al., 2006; Yap et al., 2006, 2007); yet in both cases, *CypA* fused to *TRIM5* specifically (Virgen et al., 2008).

According to a recent hypothesis, genes that are used together repeatedly and persistently in a certain context are more likely than otherwise to undergo a fusion mutation (Livnat & Papadimitriou, 2016; Livnat, 2017). In other words, it is genes that are used together that can be effectively fused together in the course of evolution for mutational reasons (henceforth the “used-fused” hypothesis; Livnat , 2017). Such genes are likely transcribed at the same time and in the same place in the nucleus—for example in transcription factories, where DNA loops bring together nearby as well as remote interacting genes (Jackson et al., 1993; Edelman & Fraser, 2012; Papantonis & Cook, 2013). According to the used-fused hypothesis, this contemporaneous, co-spatial activity causes the chromatin to be open at both loci simultaneously, brings the loci close together spatially if they are remote, and enables the generation of a gene fusion by various downstream mechanisms, such as reverse transcription of mRNAs, potentially aided by *trans*-splicing, or other mechanisms (e.g., transposable-element mediated translocation, recombination, etc.) (Livnat, 2017).

It has been furthermore argued that this hypothesis applies not only to genes that are used together in the service of germline functions, but also to genes that are used together in the service of somatic functions, because information indicating that they work together, such as shared *cis*-regulatory motifs and transcription factors that bind to them, is present in the DNA and accessible in the germline (Livnat & Papadimitriou, 2016; Livnat, 2017). Indeed, many somatic genes that appear unrelated to germline activity are regularly transcribed in the germline due to the germline-specific phenomenon known as transcriptional promiscuity (Kleene, 2005), potentially allowing them to participate in mutational mechanisms involving interactions between genes (Livnat, 2013, 2017). Thus, it has been argued that the used-fused hypothesis applies also to somatic genes *without* invoking a Lamarckian transfer of information from soma to germline (Livnat, 2017).

Although it has been known that genes that interact in one species are often fused in others (Marcotte et al., 1999; Enright et al., 1999), prior to the used-fused hypothesis, it had not been suggested that the fusion of genes that work together in evolution can be tied systematically to mutational mechanisms. Focusing not on the mechanisms of mutation origination but on the consequences of mutation, one hypothesis had suggested that the fusion of two protein domains increases their effective concentrations with respect to each other and thus allows interactions between them to evolve, and that the maintenance of the fused gene in one lineage and fission in another leads to the observed pattern (Marcotte et al., 1999). That hypothesis was criticized by Doolittle, who asked whether gene fusion actually provides a selective advantage based on effective concentrations of gene products, as fusion is not required for gene products to effectively meet (Doolittle, 1999). In the realm of cancer research, it had been argued that a pair of interacting genes expressed in the same transcription factory can undergo fusion at the RNA level through *trans*-splicing, and that this RNA fusion may be a prerequisite for cancer-causing chromosomal translocations (Gingeras, 2009). However, that hypothesis had been limited to particular mutations in cancer and had not offered to see the connection between genetic interaction and mutation as a broader phenomenon or as relevant to evolutionary change. In contrast, the used-fused hypothesis conceptualizes the connection between genetic interaction and mutation as a broad phenomenon that is part of the evolutionary process at its fundamental level and proposes a unifying framework explaining why there are parallel or recurrent gene fusions both in evolution (Carvalho et al., 2010; Livnat, 2013) and in genetic disease and cancer (Li et al., 2008; Osborne, 2014).

Here we used bioinformatic data to test the used-fused hypothesis against two main alternatives. One alternative is that fusions are driven by local transcriptional read-through or deletion mutations that affect genes that are nearby each other. Because genes that are nearby each other are also more likely to interact compared to those that are farther away (Michalak, 2008; Koonin, 2009; Ghanbarian & Hurst, 2015; Lian et al., 2018), interacting genes will be over-represented among fusions ($H_1$). To the degree that read-through or deletion mutations can generate a functional gene fusion in a single mutational event, one could then attribute fusion mutations to random mutation or accident. If so, then any correlation between gene interaction and fusion is coincidental—interactions do not drive fusion but rather interacting genes are randomly fused by virtue of their proximity. Thus, $H_1$ predicts that a correlation between gene interaction and the probability of a gene fusion mutation is coincidental, and fusion mutation is not driven by genetic interactions.

A second possibility is that gene fusion mutations occur at random between any pair of genes, but that among the pairs thus fused, those whose members had been interacting prior to fusion are more likely to end up being favored by selection ($H_2$)—because their fusion makes their interaction more effective (Marcotte et al., 1999), or because their fusion is more likely to generate a beneficial new protein. In contrast to $H_1$ described above, which focuses on adjacent or nearby genes, $H_2$ applies in principle to any pair of genes, whether nearby or distant from each other.

The used-fused hypothesis contrasts with both alternatives: it argues that genes that are used together are more likely to undergo a fusion mutation for mechanistic reasons inherent to their interaction; namely, the probability of gene fusion mutation is increased by the interaction between genes.

The three hypotheses have distinct, empirically testable consequences. $H_1$, according to which nearby genes are more likely to undergo a fusion mutation and are also (unrelatedly) more likely to interact does not offer an explanation for the recurrent fusion of the TRIM5 and CypA genes, which occurred by transposition as opposed to a read-through or deletion mutation. Generalizing from this point, if there is a correlation between interaction and fusion among distant genes, it could not be accounted for by $H_1$ alone. In addition, fusions between nearby genes do not necessarily support $H_1$: if, among different pairs of nearby genes that are of the same intra-pair distance, those that interact more tightly are more likely to undergo fusion mutation, that could not be explained by distance per se and thus by $H_1$ either.

$H_2$, on the other hand, which argues that genes fuse at random but that the fusions of those that had interacted previously are more likely to be favored by selection, applies to any pair of genes, whether nearby or distant from each other. Therefore, it could explain a correlation between gene interaction and fusion for both genes that are nearby and those that are distant from each other. However, $H_2$ would be at a disadvantage compared to $H_1$ in explaining any effect whereby proximity per se facilitates fusion, because $H_2$ ignores the obvious potential of the genomic architecture to explain such an effect through mutational considerations. Although one could combine $H_1$ and $H_2$, that would go against the principle of Occam’s razor if there is another, simpler explanation to the facts taken as a whole.

In contrast, the used-fused hypothesis makes a systematic and overarching prediction based on pre-existing interactions and thus could address in one not only all of the potential effects mentioned so far but other important ones to be discussed below. Whether two genes are nearby or distant from each other, it predicts that the more tightly they interact, the more likely they are to undergo a fusion mutation due to their interaction. Furthermore, because it involves mutational mechanisms, it would fit no less than $H_1$ a correlation between the proximity between genes and their fusion probability. Indeed, it additionally offers an explanation for why genes that are nearby each other have come to be so in the first place, as it is a straightforward extension to hypothesize that, if genes that are used together are more likely to undergo a fusion mutation, they may also be more likely to undergo a translocation mutation that will bring them into each other’s vicinity when initially distant.

Other potential consequences also bear in a critical manner on the comparison between hypotheses. While one could argue that a tendency of nearby genes to fuse, if it exists, is conceivably unrelated to their interaction ($H_1$), the same could not be argued of a similar tendency, if it exists, for genes belonging to the same topologically associating domain (TAD) to fuse: because of the inherent connection between the mechanisms of gene interaction and the mechanisms of 3D proximity (Le Dily et al., 2014; Neems et al., 2016; Tarbier et al., 2020), arguments that genes in the same TAD are more likely to undergo a fusion mutation due to their proximity in 3D would make little sense if those arguments are disconnected from gene interactions. Therefore, if genes in the same TAD are more likely to undergo a fusion mutation, that would further favor the used-fused hypothesis over $H_1$.

In addition, from the perspective of $H_2$, which relies on selection to determine the viability of fusions, no statistically significant overlap would be expected between the group of gene pairs that fuse in the course of evolution and the group of gene pairs that fuse in cancer, because the selection pressure that is acting on a gene fusion in evolution based on organismal survival and reproduction is different from the selection pressure that is acting on a gene fusion in cancer based on proliferation within an organism as a cancerous element. In contrast, if fusion mutation is mechanically limited to genes that interact tightly, then an overlap could be expected between pairs that undergo fusion in cancer and pairs that undergo fusion in evolution.

All taken together, it is clear that, even though it has been observed that genes that interact in one species are often fused in another (Marcotte et al., 1999; Enright et al., 1999), whether this fact is due to random mutation and natural selection or due to mutational mechanistic reasons, and whether such mechanistic reasons, if they exist, constitute a limited effect based on local random mutations, or apply broadly based on interactions between genes, has not been tested. Table 1 summarizes the three main hypotheses considered here and their different predictions.

**Table 1:** Alternative hypotheses on the causes of gene fusions

Hypotheses	$H_{1}$	$H_{2}$	Used-fused
	Random transriptional read-through or deletion mutations unrelated to the interactions between genes can only fuse genes that are nearby each other, and those are independently known to be more likely to interact with each other than remote genes, thus generating a correlation between interaction and fusion mutation	Genes become fused by random mutation, and among the fusions thus formed, those made by genes that had already been interacting prior to fusion are subsequently more likely to be favored by selection	Due to interaction-based mutational mechanisms, genes that interact more tightly are more likely to undergo a fusion mutation in the course of evolution
Predictions			
The more tightly genes that are nearby each other interact, the more likely they are to be fused in other species	No	Yes	﻿Yes
The more tightly genes that are remote from each other interact, the more likely they are to be fused in other species	No	Yes	﻿Yes
Among genes that are separate in one species, those that are nearby each other are more likely to be found fused in other species than those that are remote from each other	﻿Yes	Inferior to $H_{1}$ Ignoring the strong potential of mutational considerations	﻿Yes
The more frequently genes are observed together in the same TAD, the more likely they are to be found fused in other species	No	Inferior to Used-fused Ignoring the strong potential of mutational considerations	﻿Yes
Genes that are separate in humans and have become fused in the course of evolution in other species are more likely to undergo fusion in human cancers	Can only explain overlap in fusions between nearby genes	Unlikely Selection pressures differ between whole organisms and cancer	﻿Yes
Explains parallel and recurrent fusions in both evolution and disease	Inferior to Used-fused Does not apply to fusions by translocations	Inferior to Used-fused Highly unlikely for fusions by translocation and lacks mechanistic restrictions on fusion	﻿Yes

Using existing databases and several measures of interaction, including co-expression, the tendency of the two genes to be found in the same TAD, co-localization of the gene products in the cell, and semantic similarity of their associated GO terms, we compared pairs of separate human genes whose orthologs are known to be fused in other species (henceforth, “fusion-related” pairs) to random pairs of human genes, while controlling for the genomic distance between pair members, in order to examine whether the interaction between pair members is stronger in the fusion-related group. We tested this both in general as well as separately for pair members that are nearby and those that are distant from each other, by examining both pair members that are on the same and on different chromosomes as well as pair members that are on the same chromosome but at different intra-chromosomal genomic distance from each other. Second, we compared fusion-related pairs to random pairs in terms of their presence in a large database of gene pairs that undergo fusion in human cancers, again while controlling for intra-pair genomic distance.

Third, because the interpretation of these tests in terms of the used-fused hypothesis would rely on the assumption that a substantial fraction of the cases where two separate human genes have fused orthologs in other species are indeed fusion rather than fission events, we created a pipeline to identify a sample of genes that are separate in humans but whose protein products are fused in one or more of six primate species, in order to cross-validate the results using this independently obtained dataset, infer historical fusion and fission events and examine whether fission alone could account for the results. At the same time, this additional dataset allowed us to pursue an additional goal. Because the difficulty of explaining parallelism in gene fusions would be alleviated if gene interactions cause fusions, as related species share many of the same genetic interactions and therefore are expected to generate independently similar fusion mutations according to the used-fused hypothesis, observations of independent originations of the same fusions in different species would favor the used-fused hypothesis over its alternatives. Therefore, we used this independently generated dataset to examine the possibility of independent recurrence of fusions.

As predicted, fusion-related genes were more likely to interact with each other than randomized control pairs of the same genomic distance between pair members, both for pair members that are nearby and those that are distant from each other, favoring the used-fused hypothesis over $H_1$; gene pairs that had been fused in evolution were more likely than random pairs of the same intra-pair distance to be fused in human cancers, favoring the used-fused hypothesis over both $H_1$ and $H_2$; and fusion-related genes were more likely to be in the same TAD compared to random pairs of the same intra-pair distance, favoring the used-fused hypothesis over both $H_1$ and $H_2$ (Table 1). Furthermore, tissue-specific evidence from these analyses suggested that the used-fused effect applies to somatic as well as germline genes. Finally, in the dataset from six primate species, a used-fused distance-controlled effect was observed, cross-validating the results with an independently obtained dataset, and fusions dominated fissions by at least 50:1 and often recurred independently, further supporting the used-fused hypothesis.

The used-fused effect exemplifies how the causes of a mutation can be tied to its consequences via a mutational mechanism: as assumed under $H_2$, it is biologically meaningful that genes that are fused are more likely to be ones that had previously interacted, though counter $H_2$ it is not random mutation that generates this effect. Indeed, under the assumption that genes that work together tend to generate more beneficial or less disruptive fusions, the findings exemplify how mutational mechanisms affect the fitness distribution of mutation. The used-fused effect also implies that evolutionary parallelism is due not only to similar selection pressures and phenotypic effects of mutations in related species (Blount et al., 2018) but also to similar mutational tendencies (Livnat, 2013). This suggests that parallelism in gene fusion mutations may be much more extensive than previously thought and provides an explanation of the tendency of gene fusions to recur independently, both in evolution and in genetic disease and cancer. As will be discussed, the underlying concepts and results suggest that exon shuffling is initiated by the used-fused effect rather than random mutation, and that the evolution of genome organization is largely driven by mutational mechanisms rather than random mutation and random genetic drift (cf. Lynch 2007). These implications underscore the importance of studying the causes of mutation for our understanding of evolution as well as for our understanding of genetic disease and cancer.

Two further remarks are helpful. By providing evidence that the origination of fusion mutations requires an explanation other than random mutation, our results do not contradict the fact that selection may act on fusion mutations after they arise. In fact, according to the used-fused hypothesis, selection occurring over generations prior to a gene fusion mutation influences the origination of that mutation by shaping the information present in the genome, such as transcription factor binding sites, epigenetic marks and chromatin states. This information shapes the genetic interactions in the germ cells, which in turn influence fusion mutation probabilities (Livnat, 2013, 2017). Finally, only a first step in the study of the used-fused hypothesis is pursued here by examining evidence on whether used-fused mechanisms exist. Although we have outlined possibilities regarding the molecular biological nature of such mechanisms, future studies will be needed to explore those mechanisms in detail.

## Methods

### Data Collection for the Gene Interaction and Evolution-Cancer Overlap Analyses

STRING is a large database providing information on interactions between proteins, including information on gene fusions in numerous species (Szklarczyk et al., 2019). STRING treats gene fusion as any other indicator of gene interaction, and gathers these different indicators independently of each other. We used STRING to identify pairs of separate human genes whose orthologs are fused in other species (henceforth “fusion-related pairs”) and test whether the fusion-related pair members tended to interact more with each other in humans compared to randomly generated gene pairs from the human genome, while controlling for the genomic distance between pair members.

We extracted from STRING all pairs of human proteins that had a non-zero fusion score, suggesting that homologs of these proteins are fused in another or other species. Next, we mapped each pair of identified fusion-related proteins to the genes that express them to create a list of fusion-related gene pairs. Because multiple protein products can be produced by the same gene(s), we scanned the resulting list for redundancy and removed repetition, ensuring that each pair was represented only once.

#### Human Genomic Information

We used human genomic data from the NCBI repository (NCBI, 2004a) to identify locations of genes as well as to create lists of randomized control gene pairs. Because NCBI uses its own numerical identification system to provide a unique identifier (ID) for each gene (O’Leary et al., 2016; NCBI, 2004a), whereas STRING and other databases used here (see below) are based on different ID systems, we converted all identifiers to NCBI gene IDs using translation files downloaded from the NCBI repository, thus ensuring gene ID matching. In cases where non-NCBI IDs had no matching NCBI IDs or there was no one-to-one translation (multiple identifiers mapped to a single NCBI ID or vice versa), the genes were removed from analysis.

#### Creating Lists of Distance-Matched, Randomized Control Pairs

We compared the gene interactions of fusion-related pairs to those of two control groups—a “genomic control” group, where protein-coding genes from the human genome were drawn at random and paired up, and a “STRING control” group, where protein-coding genes from the subset of STRING pairs not indicated to have undergone fusion in other species were drawn at random. In both cases we controlled for the distance between pair members: pairs were drawn while matching fusion-related and control pairs in terms of the genomic distance between pair members, ensuring that each genomic distance between pair members observed in the fusion-related group was equally represented percentage-wise in each control group. For this purpose, distance was measured in terms of the number of coding genes separating between pair members. Controlling for distance is important, because the distance between genes is expected to be correlated with their co-functioning (Michalak, 2008; Koonin, 2009; Ghanbarian & Hurst, 2015; Lian et al., 2018).

To prepare the genomic control list, for each pair of fusion-related genes from STRING, we drew at random a gene from the human genome, and then paired it with a gene downstream to it found at the same distance from it as the distance between the members of the focal fusion-related STRING pair. If a partner could not be assigned (for instance because the randomly drawn gene was found at the end of a chromosome), the drawn gene was discarded and another was chosen at random from the genome. In this manner, random gene pairs were drawn without repetition 10 times for each fusion-related pair.

To prepare the STRING control, all fusion-unrelated gene pairs in the STRING database (Szklarczyk et al., 2019) were grouped by intra-pair genomic distance, and for each fusion-related gene pair, 10 fusion-unrelated STRING pairs of the same distance were drawn at random without repetition. These procedures produced control lists generally 10﻿× larger than the list of fusion-related pairs: in cases where we could not obtain 10 random pairs per fusion-related pair, we used all available pairs for analysis.

A positive result using the genomic control would mean that fusion-related pair members are more likely to interact with each other compared to random pairs of the same intra-pair genomic distance, and a positive result using the STRING control would mean that fusion-related pair members interact more tightly with each other compared to pair members of the same intra-pair genomic distance that are known to interact with each other but are not known to have fused in other species.

#### Distance Groups

We extracted the genomic positions of all human genes from the human gene-feature table downloaded from the NCBI repository (O’Leary et al., 2016). Using this data, we first made a high-level separation between gene pairs whose members are on the same chromosome (“same chromosome” group) and pairs whose members are on different chromosomes (“different chromosome” group). Then, we found how many protein-coding genes are found between genes in each of the pairs and divided the same-chromosome group into four different distance categories: pairs whose members are separated by *i*) no coding genes (SC_0), *ii*) 1–99 coding genes (SC_1–99), *iii*) 100–499 coding genes (SC_100–499) and *iv*) 500 coding genes or more (SC_500+) (see Supplemental Text S1).

### Gene Interaction Analyses

To examine whether fusion-related gene pair members interact with each other more tightly than members of randomized, distance-matched control pairs, we used several different measures of interaction, including co-expression, the tendency of the two genes to be found in the same TAD, co-localization of the gene products in the cell, and semantic similarity of their associated GO terms.

#### Co-expression Analysis

To test whether fusion-related gene pairs are more highly coexpressed than control pairs, we used COXPRESdb (Obayashi et al., 2018, 2019). COXPRESdb provides four databases of human gene co-expression (Obayashi et al., 2019), which differ in the platform used to obtain gene expression data and in the methods used to compute the co-expression scores from the raw data (Obayashi et al., 2018). We used all four to examine the consistency of the results across them. We extracted the co-expression score for each pair of fusion-related and each pair of control genes using a custom Perl script and then tested whether fusion-related gene pairs are more highly co-expressed than random pairs using the one-sided Mann–Whitney–Wilcoxon test (all tests were performed with R) (R Core Team, 2019)

To test whether the observed correlation between gene fusion and co-expression applies to both germline and somatic tissues, we downloaded from the GTEx portal (Lonsdale et al., 2013) a database containing human gene expression data (GTExv7; GTEx, 2013) based on samples taken from different tissues and different patients. These data varied across tissues in terms of the number of donors represented in each tissue (Table S3). We excluded tissues with less than ten data-points (endocervix, ectocervix, fallopian tubes) from analysis. Next, we divided the remaining data into subsets, each containing expression data from a single tissue from multiple patients. We then analyzed the co-expression of gene pair members using two methods: (1) We obtained the co-expression across patients for each gene pair within each tissue separately and then averaged it across all tissues. (2) For each gene, we averaged the expression value per tissue across patients and then obtained the co-expression across tissues. In both cases, we used the Spearman $\rho$ coefficient of the correlation between the expression values of pair members as a measure of coexpression. To measure co-expression in the soma only, we excluded expression data from the testis and ovary tissues from analysis.

#### Topologically Associating Domain (TAD) Presence Analysis

A topologically associating domain (TAD) is a region in the genome whose DNA sequences interact physically preferentially with each other and are found in close proximity to each other in 3D space (Dekker & Heard, 2015). We used a database of human genome TAD coordinates downloaded from the 3D Genome Browser (Wang et al., 2018) to investigate whether fusion-related pair members tend to be found in the same TAD more frequently than members of random pairs while controlling for the 2D genomic distance between pair members. Since no information is provided by the aforementioned database on interactions across different chromosomes, for the TAD analysis we examined only same-chromosome pair members.

The data consisted of several independent lists resulting from different experiments and studies (Wang et al., 2018). We identified the boundaries of the genes in each pair using data from the gene feature table of *H. sapiens* downloaded from the NCBI repository (O’Leary et al., 2016). Next, for each gene pair we determined the number of individual TAD coordinate lists in which both genes were found in the same TAD. A gene was considered to be present in a TAD if the entirety of it was included in that TAD. Finally, we examined whether fusion-related gene pairs are found in the same TAD across a significantly larger number of TAD lists than control pairs using a one-sided Mann–Whitney–Wilcoxon test.

#### Co-localization Analysis

To compare the extent to which protein products of pair members localize to the same cellular compartment between the fusion-related pairs and control pairs, we used a database of protein sub-cellular localization predicted by WoLF PSORT, downloaded from the COXPRESdb portal (Obayashi et al., 2018; Horton et al., 2007). We marked a gene pair as co-localized if any of the protein products of one gene and any of the protein products of the other were associated with the same cellular compartment term, and then tested whether the proportion of co-localized gene pairs was higher among fusion-related than control pairs using a one-sided Fisher exact test.

#### Analysis of GO Terms’ Semantic Relatedness

Gene Ontology (GO) terms represent highly structured, common and well-defined vocabulary describing the roles of genes and gene products in any organism (Hill et al., 2002; GO Consortium, 2008). Their analyses are used to study functional relationships between genes and cluster genes based on their functional similarities (Zhao & Wang, 2018). We downloaded from the NCBI repository (NCBI, 2004a) the ‘Gene2GO’ list, which associates GO terms with genes, and used a custom Perl script to extract GO terms for the fusion-related and control gene lists. Next, we used GOGO, a program for measuring semantic similarity of GO terms (Zhao & Wang, 2018), to obtain the similarity score of GO terms for the genes in each pair. Finally, we tested whether those similarity scores were higher in the fusion-related than in the control gene pairs using a one-sided Mann–Whitney–Wilcoxon test.

GO terms contain three main categories: Biological Process (BP), Molecular Function (MF) and Cellular Component (CC). Because not all of the genes had associated GO terms for all three categories, we conducted the analysis separately for each of these three main categories. Accordingly, the control lists for these analyses were also created separately for each category.

### Analysis of Presence in the Database of Cancer-Related Fusions

To test for a potential overlap between gene fusions occurring in evolution and those occurring in cancer, we downloaded data of gene fusions observed in cancers (whether DNA fusion or fusion of transcripts) from the Cosmic (Tate et al., 2018) and Fusion-GDB (Kim & Zhou, 2018, 2019) portals and combined it into a single list. We used a custom Perl script to calculate the number of pairs that overlap between the lists of fusion-related pairs from STRING and pairs involved in cancer fusions, as well as the number of pairs that overlap between the control lists and the latter, and then tested whether the proportion of overlapping pairs was significantly higher among evolutionary fusion-related pairs than among control pairs using a one-sided Fisher exact test.

In the cancer databases used in this study, genes were indicated by their symbol rather than numerical gene ID (NCBI or Ensembl). To convert the gene IDs into symbols, we used the “geneinfo” table downloaded from the NCBI repository (NCBI, 2004a). When checking for pair presence within the cancer databases, we used all symbols (official symbol and common synonyms) associated with a particular gene in the “geneinfo” table.

### Analysis of Fusions in Six Primate Species

#### Primate Datasets and Identification of Potential Primate-Fusion–Related Human Genes

We analyzed six primate species for the presence of fusion proteins: *Pan troglodytes* (Chimpanzee, assembly: GCF002880755.1), *Gorilla gorilla gorilla* (Gorilla, assembly: GCF_000151905.2), *Macaca nemestrina* (Pig-tailed macaque, assembly: GCF_000956065.1), *Aotus nancymaae* (Owl monkey, assembly: GCF_000952055.2), *Callithrix jacchus* (Common marmoset, assembly: GCF_000004665.1) and *Microcebus murinus* (Mouse lemur, assembly: GCF_000165445.2). All of the protein sequence datasets were downloaded from the NCBI repository (O’Leary et al., 2016; NCBI, 2004b). The analyzed protein sequences included all of the protein products annotated on the genome assembly of the species studied.

The human protein dataset was compared to the protein datasets of each of the six primate species using FASTA (Pearson & Lipman, 1988). A pair of human genes was considered to be “fusion-related” if any of the protein products of each gene mapped to a single primate protein above the following thresholds: (1) The alignment length between the primate protein and each human protein mapping to it was at least 20 amino acids (see Supplemental Text S2). (2) Proteins from the two different human genes mapped to the same primate protein with no more than a 5 amino acid overlap between their respective alignment regions; the alignment region between a human and a primate protein was defined as the region covered by all of the isoforms of the given human protein mapping to the primate protein. (3) The e-value of each alignment was at most 0.1. (4) The similarity between the aligned regions in the human and primate proteins was above the identity threshold for the given primate species (see Supplemental Text S3). If proteins from two distinct human genes mapped to the same primate protein within the overlap limits, but a protein from another human gene aligned to the same primate protein within the identity and e-value thresholds such that its aligned region included those of the other proteins, the fusion candidate gene was discarded.

#### Identification of Likely Human Homologs of Primate Fused Proteins

We divided the list of all human proteins aligning to the fused proteins in each studied primate species into two categories. The first included all cases where proteins from only two distinct human genes aligned to a single primate protein. These two genes were then considered to be the most likely human homologs of the primate gene. The second category included all cases where proteins from more than two human genes aligned to a single primate protein within the thresholds outlined above. The most likely homologs of the fused primate protein among the different human genes were then identified by a manual similarity analysis (Supplemental Text S4).

#### Analysis of Genomic Distances Between Primate Fusion﻿–Related Pair Members

We repeated the analysis of genomic distance between fusion-related pair members in the STRING database for the identified primate fusion–related human genes. The genomic positions of all human genes were extracted from the human gene feature table downloaded from NCBI (2004b) and the distance between the genes in each pair was measured as before by the number of protein-coding genes separating them. The gene pairs were divided as before into same- and different-chromosome groups and into four within-chromosome distance categories: SC_0, SC_1–99, SC_100–499, SC_500+.

#### Analysis of Presence of Primate Fusion﻿–Related Gene Pairs in the STRING Database

Presence of a gene pair in the list of gene pairs for which interactions other than fusion are indicated by STRING serves as gross-level evidence of interaction between the pair members which encompasses various possible types of interaction. We extracted from the STRING database all pairs of proteins for which interaction indicators other than fusion exist and considered a pair of primate fusion–related human genes to be present within this STRING group if any of the protein products of one pair member was paired with any of the protein products of the other pair member in this group. Finally, we created for the primate fusion﻿–related pairs a distance-matched genomic control group in the same way as before, and tested whether the protein products of the primate fusion﻿–related pairs are more likely to appear in the STRING-extracted group than the protein products of the distance-matched control pairs using a one-sided Fisher exact test.

#### Phylogenetic Analysis of Primate Fused Proteins

For each pair of fusion-related human genes, the primate lineages in which their fusion was found were noted, and the number of times that fusion or fission events occurred independently were inferred according to the standard phylogenetic parsimony method (Fitch, 1971). If the observed distribution could be explained by a number of distinct scenarios involving an equal number of independent events, all of these scenarios were noted.

## Results

### Gene Interaction Analyses Results

Across four measures of gene interaction—co-expression, presence of the two genes in the same TAD, co-localization of the gene products in the cell, and semantic similarity of their associated GO terms—we compared fusion-related pairs (pairs of separate human genes whose orthologs are known to be fused in other species) to randomized, distance-matched control pairs, of both the genomic and STRING kinds. Specific results are described below.

#### Fusion-Related Pair Members are More Highly Co-expressed than Members of Distance-Matched Random Pairs

Consistently across the four COXPRESdb databases, co-expression was significantly higher in the fusion-related pairs than in both the genomic and STRING control pairs. This pattern was significant both for pair members within the same chromosome (*p* < 2.20E−16 in both the genomic and STRING controls; one-sided MW test) and for pair members on different chromosomes (*p* < 2.20E−16, genomic and *p* $\le$ 2.13E−02, STRING control; one-sided MW test) (Table 2).

**Table 2:** Co-expression comparisons between fusion-related and control gene pairs using COXPRESdb

Distance^a^	Genomic control			STRING control		
	Group size^b^	*p*-value^c^	W^c^	Group size^b^	*p*-value^c^	W^c^
Hsa-m2-v18-09						
All_pairs	10438–104380	< 2.20E−16	3.87E+08	10438﻿–103504	< 2.20E−16	4.74E+08
Same chromosome	2573–25730	< 2.20E−16	1.75E+07	2573﻿–24854	< 2.20E−16	2.05E+07
SC_0	746–7460	3.68E−09	2.43E+06	746﻿–6597	8.05E−03	2.33E+06
SC_1-99	1412–14120	< 2.20E−16	2.87E+06	1412﻿–14120	< 2.20E−16	3.94E+06
SC_100-499	269–2690	3.71E−09	2.85E+05	269﻿–2690	8.48E−01	3.76E+05
SC_500+	146–1460	6.60E−06	8.33E+04	146﻿–1447	3.66E−01	1.04E+05
Different chromosomes	7865–78650	< 2.20E−16	2.28E+08	7865﻿–78650	< 2.20E−16	2.90E+08
Hsa-m-v18-10						
All pairs	9453–94530	< 2.20E−16	3.41E+08	9453﻿–93472	< 2.20E−16	4.12E+08
Same chromosome	2279–22790	< 2.20E−16	1.55E+07	2279﻿–21732	< 2.20E−16	1.78E+07
SC_0	719–7190	1.22E−10	2.22E+06	719﻿–6145	1.87E−03	2.06E+06
SC_1-99	1181–11810	< 2.20E−16	2.71E+06	1181﻿–11810	< 2.20E−16	3.67E+06
SC_100-499	259–2590	1.04E−15	2.35E+05	259﻿–2590	2.01E−01	3.25E+05
SC_500+	120–1200	2.94E−04	5.83E+04	120﻿–1187	5.96E−01	7.22E+04
Different chromosomes	7174–71740	< 2.20E−16	2.00E+08	7174﻿–71740	2.13E−02	2.54E+08
Hsa-r-v18-12						
All_pairs	9336–93360	< 2.20E−16	2.97E+08	9336﻿–92082	< 2.20E−16	3.74E+08
Same chromosome	2292–22920	< 2.20E−16	1.26E+07	2292﻿–21642	< 2.20E−16	1.45E+07
SC_0	688–6880	2.15E−10	2.03E+06	688﻿–5616	2.28E−03	1.80E+06
SC_1–99	1238–12380	< 2.20E−16	1.75E+06	1238﻿–12380	< 2.20E−16	2.51E+06
SC_100-499	241–2410	7.01E−16	2.00E+05	241﻿–2410	1.68E−01	2.79E+05
SC_500+	125–1250	5.59E−07	5.75E+04	125﻿–1236	4.95E−01	7.72E+04
Different chromosomes	7044–70440	< 2.20E−16	1.75E+08	7044﻿–70440	< 2.20E−16	2.34E+08
Hsa-u-v18-12						
All pairs	10,372–103720	< 2.20E−16	3.75E+08	10372﻿–102460	< 2.20E−16	4.69E+08
Same chromosome	2552–25520	< 2.20E−16	1.63E+07	2552﻿–24260	< 2.20E−16	1.91E+07
SC_0	776–7760	4.30E−14	2.52E+06	776﻿–6513	3.17E−04	2.34E+06
SC_1-99	1371–13710	< 2.20E−16	2.46E+06	1371﻿–13710	< 2.20E−16	3.55E+06
SC_100-499	266–2660	< 2.20E−16	2.41E+05	266﻿–2660	1.60E−01	3.41E+05
SC_500+	139–1390	5.99E−08	7.03E+04	139﻿–1377	3.46E−01	9.38E+04
Different chromosomes	7820–78200	< 2.20E−16	2.18E+08	7820﻿–78200	2.43E−14	2.90E+08

Tests were performed for each of the four different databases of human gene co-expression in COXPRESdb

^a^Distance is measured by the number of protein-coding genes separating between the members of the analyzed gene pair

^b^Number of gene pairs in the fusion-related (left) and control group (right). The control group represents a 10× larger group than the fusion-related group. If for a certain distance group the number of possible control pairs was smaller than 10× the number of fusion-related pairs, all available control pairs were used for the analysis

^c^One-sided Mann–Whitney test

The co-expression differences between fusion-related and control pairs as a function of the more fine-grained different categories of genetic distance within chromosome are shown in Table 2. Though these groups have smaller sample sizes, the pattern is mostly consistent across all groups. In addition, fusion-related genes that are found in close proximity to each other (i.e., with less than 100 genes separating them) are significantly more highly co-expressed than random control pairs with the same genomic distance between them (genomic control) and are furthermore more highly co-expressed than fusion-unrelated genes that are known to interact and are separated by the same distance (STRING control). Some of the fine-grained comparisons within the intra-chromosomal distance groups in the STRING control case are not individually significant. However, because of the strongly significant results that are obtained for the larger group of different chromosome pair members, which demonstrate that the effect applies to genes that are distant from each other, and because the STRING control is a more stringent one (as it includes pairs for which indicators of interaction already exist) the non-significance in these groups is likely due to their limited group sizes. Consistent with this interpretation, the results in the small within-chromosome STRING control groups are mostly in the expected direction.

Because the gene pairs studied have not been selected based on tissue and because there are many more somatic than germline tissues, one may expect that the results are not limited to genes that serve germline functions only. To confirm this, we repeated the co-expression analysis while excluding expression data from both the testis and ovary tissues using the GTEx database (Lonsdale et al., 2013; GTEx, 2013), which provides per-tissue gene expression data (Table S1). Results show a similar pattern as those described above, demonstrating that the correlation between gene fusion and co-expression is not driven by germline tissues specifically.

#### Fusion-Related Pair Members are More Often Found in the Same TAD than Members of Distance-Matched Random Pairs

Examining all of the same-chromosome gene pairs together, we found that fusion-related pair members are more often found in the same TAD than control pair members (*p* < 2.20E−16 in both the genomic and STRING control cases; one-sided MW test) (Table 3). As noted, the data only lists TADs as within-chromosome regions, rendering the analysis of different chromosomes inapplicable. The differences in the within-chromosome analyses are mainly driven by the SC_1–99 group (*p* < 2.20E−16 for both the genomic and STRING controls; one-sided MW test). Smaller yet still significant differences in same-TAD presence exist in the neighboring genes group (SC_0) (*p* = 1.07E−02 genomic and *p* = 2.54E−03 STRING; one-sided MW test). Although small, this last effect is notable given that neighboring genes are likely to be present in the same TAD due to their proximity to each other alone, thus reducing the potential for finding a difference in same-TAD-presence between fusion-related and control neighboring genes groups to begin with.

**Table 3:** Same-TAD presence comparisons between the fusion-related and control gene pairs

Distance^a^	Genomic control			STRING control		
	Group size^b^	*p*-value^c^	*W*^c^	Group size^b^	*p*-value^c^	*W*^c^
All pairs	2747–27470	< 2.20E−16	4.65E+07	2747–26368	< 2.20E−16	4.47E+07
Same chromosome	2747–27470	< 2.20E−16	4.65E+07	2747–26368	< 2.20E−16	4.47E+07
SC_0	805–8050	1.07E−02	3.40E+06	805–6961	2.54E−03	2.97E+06
SC_1–99	1495–14950	< 2.20E−16	1.63E+07	1495–14950	< 2.20E−16	1.58E+07
SC_100–499	281–2810	2.94E−01	3.96E+05	281–2810	2.09E−01	3.97E+05
SC_500+	166–1660	1.00E+00	1.38E+05	166–1647	1.00E+00	1.37E+05

^a^Distance is measured by the number of protein-coding genes separating between the members of the analyzed gene pair

^b^Number of gene pairs in the fusion-related (left) and control group (right). The control group represents a 10× larger group than the fusion-related group. If for a certain distance group the number of possible control pairs was smaller than 10× the number of fusion-related pairs, all available control pairs were used for the analysis

^c^One-sided Mann–Whitney test

Because of the size distribution of TADs (Rao et al., 2014; Muro et al., 2019; Long et al., 2022), pair members in the larger distance categories, namely the SC_100–499 and SC_500+ groups, are likely too far apart from each other to be present in the same TAD in most if not all cases. Consistently, we found no significant differences for these categories.

#### Co-localization is Higher for Fusion-Related Pair Members than for Members of Distance-Matched Random Pairs

Examining the extent of co-localization of pair members’ protein products, co-localization was significantly higher in the fusion-related than control pairs for both the same chromosome and different chromosome groups (*p* < 2.20E−16 both genomic and STRING; one-sided Fisher exact test) (Table 4). Further analysis of the same-chromosome group reveals that the higher significance of co-localization of fusion-related genes is mainly driven by non-neighboring genes, in particular by the SC_1–99 (*p* < 2.20E−16 both genomic and STRING; one-sided Fisher exact test) and SC_500+ (*p* $\le$ 4.83E−16 genomic and *p* $\le$ 1.94E−06 STRING; one-sided Fisher exact test) groups (SC_100–499 showed high significance only for the genomic control group). This pattern may be attributed to a ceiling effect whereby neighboring genes are already highly enriched for similar features (Michalak, 2008; Koonin, 2009; Ghanbarian & Hurst, 2015; Lian et al., 2018). Thus, co-localization of pair members’ gene products is generally significantly higher for fusion-related than control pairs, both for the same-chromosome and different-chromosome groups.

**Table 4:** Co-localization comparisons between fusion-related and control gene pairs using the Psort database from the COXPRESdb portal

Distance^a^	Genomic control				STRING control			
	Group size^b^	Number of positives^c^	*p*-value^d^	Odds ratio^d^	Group size^b^	Number of positives^c^	*p*-value^d^	Odds ratio^d^
All pairs	10747–107470	7344–52454	< 2.20E−16	2.263	10747–106466	7344–64401	< 2.20E−16	1.410
Same chromosome	2644–26440	1979–13605	< 2.20E−16	2.807	2644–25436	1979–14960	< 2.20E−16	2.084
SC_0	761–7610	437–4263	2.40E−01	1.059	761–6620	437–3852	6.71E−01	0.969
SC_1–99	1456–14560	1245–7316	< 2.20E−16	5.841	1456–14560	1245–8519	< 2.20E−16	4.184
SC_100–499	272–2720	172–1289	3.88E−07	1.909	272–2720	172–1631	1.62E−01	1.148
SC_500+	155–1550	125–737	4.83E−16	4.593	155–1536	125–958	1.94E−06	2.513
Different chromosomes	8103–81030	5365–38849	< 2.20E−16	2.128	8103–81030	5365–49441	< 2.20E−16	1.252

^a^Distance is measured by the number of protein-coding genes separating between the members of the analyzed gene pair

^b^Number of gene pairs in the fusion-related (left) and control group (right). The control group represents a 10× larger group than the fusion-related group. If for a certain distance group the number of possible control pairs was smaller than 10× the number of fusion-related pairs, all available control pairs were used for the analysis

^c^Number of co-localized pair members from the fusion-related group (left) and the control group (right)

^d^One-sided Fisher exact test

#### GO Terms are More Similar Between Fusion-Related Pair Members than Between Members of Distance-Matched Random Pairs

Finally in these interaction analyses, we found a significantly higher semantic similarity of GO terms in the fusion-related than control pairs when examining the entire group of same-chromosome pair members (*p* < 2.20E−16 both genomic and STRING; one-sided MW test), as well as the group of different-chromosome pair members (*p* < 2.20E−16 genomic and *p* $\le$ 1.47E−07 STRING; one-sided MW test) for all three main GO categories: Biological Process, Molecular Function and Cellular Component (Table 5).

**Table 5:** GO term semantic similarity comparisons between fusion-related and control gene pairs

Distance^a^	Genomic control			STRING control		
	Group size^b^	*p*-value^c^	W^c^	Group size^b^	*p*-value^c^	W^c^
Biological process						
All_pairs	10389–103890	< 2.20E−16	7.46E+08	10389–102937	< 2.20E−16	5.88E+08
Same chromosome	2459–24590	< 2.20E−16	4.31E+07	2459–23637	< 2.20E−16	3.72E+07
SC_0	661–6610	9.23E−01	2.11E+06	661–5671	1.00E+00	1.71E+06
SC_1–99	1380–13800	< 2.20E−16	1.59E+07	1380–13800	< 2.20E−16	1.45E+07
SC_100–499	261–2610	< 2.20E−16	4.74E+05	261–2610	5.48E−02	3.61E+05
SC_500+	157–1570	< 2.20E−16	1.72E+05	157–1556	5.58E−02	1.32E+05
Different chromosomes	7930–79300	< 2.20E−16	4.30E+08	7930–79300	1.47E−07	3.25E+08
Molecular function						
All pairs	10491–104910	< 2.20E−16	7.08E+08	10491–103779	< 2.20E−16	6.03E+08
Same chromosome	2473–24730	< 2.20E−16	4.23E+07	2473–23599	< 2.20E−16	3.78E+07
SC_0	674–6740	9.54E−01	2.18E+06	674–5624	9.99E−01	1.76E+06
SC_1–99	1380–13800	< 2.20E−16	1.55E+07	1380–13800	< 2.20E−16	1.47E+07
SC_100–499	261–2610	1.71E−08	4.11E+05	261–2610	4.34E−01	3.43E+05
SC_500+	158–1580	6.03E−16	1.73E+05	158–1565	1.34E−05	1.49E+05
Different chromosomes	8018–80180	< 2.20E−16	4.04E+08	8018–80180	3.32E−10	3.35E+08
Cellular component						
All_pairs	10662–106620	< 2.20E−16	8.02E+08	10662–105590	< 2.20E−16	6.60E+08
Same chromosome	2574–25740	< 2.20E−16	4.69E+07	2574–24710	< 2.20E−16	4.12E+07
SC_0	722–7220	2.65E−01	2.64E+06	722–6204	8.67E−01	2.18E+06
SC_1–99	1427–14270	< 2.20E−16	1.67E+07	1427–14270	< 2.20E−16	1.55E+07
SC_100–499	265–2650	< 2.20E−16	4.64E+05	265–2650	1.89E−01	3.63E+05
SC_500+	160–1600	< 2.20E−16	1.82E+05	160–1586	3.50E−04	1.47E+05
Different chromosomes	8088–80880	< 2.20E−16	4.62E+08	8088–80880	< 2.20E−16	3.69E+08

^a^Distance is measured by the number of protein-coding genes separating between the members of the analyzed gene pair

^b^Number of gene pairs in the fusion-related (left) and control group (right). The control group represents a 10× larger group than the fusion-related group. If for a certain distance group the number of possible control pairs was smaller than 10× the number of fusion-related pairs, all available control pairs were used for the analysis

^c^One-sided Mann–Whitney test

Further analysis of the same-chromosome group revealed a similar pattern (Table 5), similar also to results from the co-localization analysis (Table 4). Significant differences between the fusion-related and control pairs were found for all three main GO categories in the SC_1–99 group (*p* < 2.20E−16, both genomic and STRING; one-sided MW test). In the SC_100–499 group, significant differences were found only in the genomic control case, in all three GO categories (*p* $\le$ 1.71E−08; one-sided MW test). In the SC_500+ group, significant differences were found in all three GO categories in the genomic control case and in the MF and CC categories in the STRING control case (*p* $\le$ 6.03E−16 and *p* $\le$ 3.50E−04 resp.; one-sided MW test). For the SC_0 group, no significant differences were found in either the genomic or STRING controls in any of the GO categories, likely due to the same ceiling effect whereby neighboring genes are already highly enriched for similar features (Michalak, 2008; Koonin, 2009; Ghanbarian & Hurst, 2015; Lian et al., 2018).

Thus, the semantic similarity between the GO terms associated with pair members is generally significantly higher in the fusion-related than control pairs, both for same-chromosome and different-chromosome pair members. For pair members on the same chromosome, these differences tend to be found in more distant pair members.

#### Summary of Gene Interaction Analyses

Consistently across all measures, analyses comparing fusion-related to randomized control pairs based on all pairs, the same chromosome group and the different chromosome group (where applicable) are all individually significant for both the genomic and STRING controls, and the preponderance of finer-scale, within-chromosome analyses, which were based on smaller sample sizes, are generally in the same direction. These results show that genes that are separate in humans but fused in non-human species are more likely to interact with each other compared to random pairs while controlling for the distance between pair members, both for genes that are nearby and those that are distant from each other. The fact that significant results are obtained even for the STRING control group and sometimes even in the smaller, within chromosome categories attests to the strength of the effect. In multiple cases, fusion-related genes are seen to interact more tightly with each other even compared to gene pairs whose members are already known to interact.

Finally, considering that for the same-chromosome groups, a greater range of distances between pair members increases the number of potential fusion-related pairs, and that the different chromosome group includes many more potential fusion-related pairs than the same chromosome group, the actual group sizes obtained suggest that the closer two genes are to each other on the genome, the more likely they are to be fused in other species.

### Evolution-Cancer Overlap: Fusion-Related Gene Pairs are More Highly Represented Than Distance-Matched Random Gene Pairs in the List of Human Cancer Gene Fusions

Results showed that cancer databases are enriched for evolutionary fusion-related gene pairs compared to control pairs for both the genomic and STRING controls both using all pairs combined (*p* = 3.97E−07 genomic and *p* = 1.57E−04 STRING; one-sided Fisher exact test) and for the same-chromosome group (*p* = 1.89E−06 genomic and *p* = 7.87E−04 STRING; one-sided Fisher exact test) (Table 6). For the different-chromosome group, the overlap was significantly larger in the fusion-related than in the control pairs in the genomic control (*p* = 8.89E−04, one-sided Fisher exact test), whereas in the more conservative STRING control it was not significant though in the expected direction (OR ${\sim }1.58$). Further analysis of the same-chromosome group revealed that its significance is driven by neighboring (SC_0) pair members (*p* = 1.68E−07 genomic and *p* = 1.17E−04 STRING; one-sided Fisher exact test), likely due to the much smaller numbers of observations of overlap both for the fusion-related pairs and for the control pairs in the larger intra-chromosomal distance groups (Table 6). Overall, these results demonstrate a statistically significant overlap between evolutionarily fusion-related gene pairs and pairs fused in human cancers. Table 7 includes a summary of the gene interaction and cancer-overlap results.

**Table 6:** Comparisons between fusion-related and control gene pairs in terms of their presence in cancer fusion databases

Distance^a^	Genomic control				STRING control			
	Group size^b^	Number of positives^c^	*p*-value^d^	Odds ratio^d^	Group size ^b^	Number of positives^c^	*p*-value^d^	Odds ratio^d^
All pairs	11291–112910	168–1091	3.97E−07	1.548	11291–111688	168–1220	1.57E−04	1.368
Same chromosome	2761–27610	162–1083	1.89E−06	1.527	2761–26388	162–1182	7.87E−04	1.329
SC_0	817–8170	126–769	1.68E−07	1.755	817–6961	126–757	1.17E−04	1.494
SC_1–99	1497–14970	35–310	2.71E−01	1.132	1497–14970	35–420	8.73E−01	0.829
SC_100–499	280–2800	0–4	1.00E+00	0.000	280–2800	0–5	1.00E+00	0.000
SC_500+	167–1670	1–0	9.09E−02	Inf	167–1657	1–0	9.16E−02	Inf
Different chromosomes	8530–85300	6–8	8.89E−04	7.505	8530–85300	6–38	2.07E−01	1.579

^a^Distance is measured by the number of protein-coding genes separating between the members of the analyzed gene pair

^b^Number of gene pairs in the fusion-related (left) and control group (right). The control group represents a 10﻿× larger group than the fusion-related group. If for a certain distance group the number of possible control pairs was smaller than 10﻿× the number of fusion-related pairs, all available control pairs were used for the analysis

^c^Number of pairs from the fusion-related (left) and control (right) groups that have been observed to fuse in human cancers

^d^One-sided Fisher exact test

**Table 7:** Summary of statistical tests for fused genes

	All pairs	Different chromosome	Same chromosome (overall)	Same chromosome (subdivisions)	
Co-expression					
Genomic control	****	﻿****	﻿****	0	﻿***
				1–99	﻿****
				100–499	﻿****
				500+	﻿**
STRING control	﻿****	﻿****	﻿*	0	﻿*
				1–99	﻿****
				100–499	
				500+	
TAD					
Genomic control	﻿****	N/A	﻿****	0	﻿*
				1–99	﻿****
				100–499	
				500+	
STRING control	﻿****	N/A	﻿****	0	﻿*
				1–99	﻿****
				100–499	
				500+	
Co-localization					
Genomic control	﻿****	****	****	0	
				1–99	****
				100–499	**
				500+	****
STRING control	****	****	****	0	
				1–99	****
				100–499	
				500+	**
GO terms					
Genomic control	****	****	****	0	
				1–99	****
				100–499	***
				500+	***
STRING control	****	**	**	0	
				1–99	****
				100–499	
				500+	** (for MF and CC)
Cancer-related					
Genomic control	**	*	**	0	**
				1–99	
				100–499	
				500+	
STRING control	**		*	0	**
				1–99	
				100–499	
				500+	

Summary of statistical significance in the co-expression, same-TAD presence, co-localization, GO terms and cancer fusion overlap analyses

*p < 5E−02; **p < 5E−04; ***p < 5E﻿−08; ****p < 5E−16

### Phylogenetic Data from Six Primate Species

We conducted a final set of analyses in order to infer historical fusion and fission events and their potential independent recurrence as well as to examine the effect again with an independent set of fusion-related pairs obtained using a pipeline for identifying pairs of genes that are separate in humans and are fused in other primates. Because the list of fusion-related pairs resulting from applying this alternative, stringent method to a small sample of species is orders of magnitude shorter than that extracted from the large STRING database, most analyses performed on the STRING list could not be effectively repeated here. However, despite the drastically reduced group size, an overall, gross-level analysis of the primates pair data is significant and consistent with the STRING-based analyses: We compiled the list of all protein pairs that have evidence for interaction other than fusion in STRING. Considering the presence of protein pairs in this list as a gross-level indicator of interaction between the corresponding genes—an indicator which includes many possible types of interaction—we found that fusion-related gene pair members are significantly more likely to interact with each other than distance-matched control pairs (Table 8), providing cross-validation for the STRING-fusion–based results (while the latter relied on fusion information from STRING, the primate-fusion analysis relies on fusion data obtained independently).

**Table 8:** Analysis of differences in STRINGdb presence between the primate fusion﻿–related and genomic control gene pairs using a one-tailed Fisher exact test

Distance^a^	Genomic control			
	Total^b^	Number of positives^c^	*p*-value^d^	Odds ratio^d^
All pairs	125–1250	67–458	1.75E−04	2.00
Same chromosome	98–980	62–452	8.41E−04	2.01
SC_0	73–730	49–397	2.38E−02	1.71
SC_1–99	19–190	12–54	3.01E−03	4.28
SC_100–499	4–40	1–1	1.75E−01	11.40
SC_500+	2–20	0–0	1.00E+00	0.00
Different chromosomes	27–270	6–6	1.36E−03	9.84

^a^Distance is measured by the number of protein coding genes separating genes in the analyzed pair

^b^Number of genes in the fusion-related (left) and control group (right). The control group represents a 10﻿× larger group than the fusion-related group. If for a certain distance group the number of possible control pairs was smaller than 10﻿× the number of fusion-related pairs, all available control pairs were used for the analysis

^c^Number of gene pairs present in STRINGdb from the primate fusion gene group (left) and the genomic control group (right)

^d^One-sided Fisher exact test

**Fig. 1:**
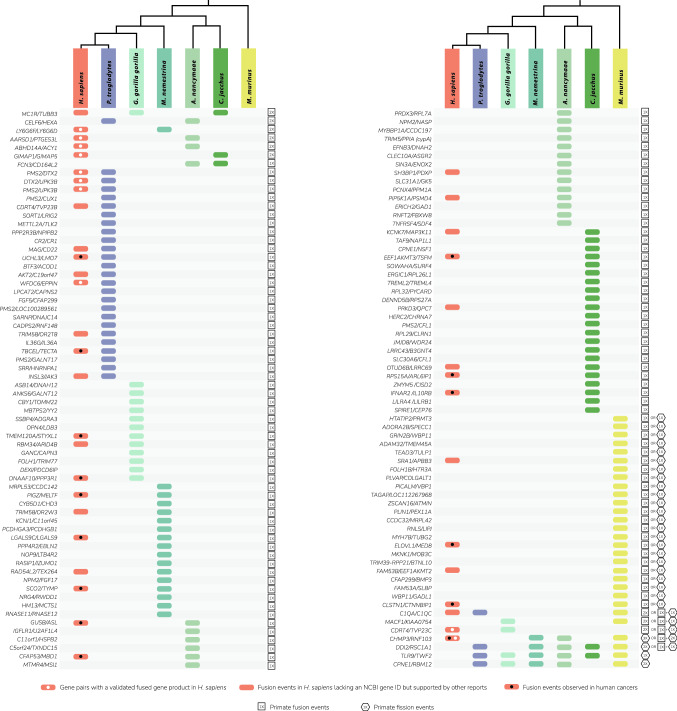
Fused genes in primate species. The names of human genes whose protein products were found to map to a protein product of a single gene in one or more of the six primate species are listed on the left in each panel. Fusions across the six species are shown below the cladogram (cladogram based on Perelman et al. 2011) for each gene pair. Gene pairs showing fusions in *H. sapiens* that are supported by scientific reports are marked by rounded rectangles; pairs with a validated fused gene product in *H. Sapiens* (i.e., with an assigned NCBI gene ID) are marked by rounded rectangles with a white dot; and fusions observed in human cancers are marked by rounded rectangles with a black dot (for human gene fusion references, see Table S2). The right column in each panel shows the minimal number and type of events (historical fusion or fission events) that may account for the observed distribution of each fusion across species under the standard phylogenetic parsimony argument, taking into account both primate fusions and human fusions that have an NCBI gene ID. Overall, with the exception of the three fusion-related pairs *PMS2/DTX2*, *DTX2/UPK3B* and *PMS2/UPK3B*, which are part of a *DTX2P1-UPK2BP1-PMS2P11* read-through pseuodogene, a total of 38 gene-pairs that are fused in at least one of the six primate species show evidence for independent fusion in humans

Next, the primate data allowed us to obtain a phylogenetic view of fusions (Fig. 1). Using the standard parsimony argument to infer ancestral events of fusion and fission, we found that the former dominate the latter ${\sim }50:1$ in this dataset (Fig. 1).

Results from a literature search on the pair members and their fusions are shown in Table S2. Consistent with the STRING-based analyses, some of the pairs (15/132) appear in human cancers. For others, evidence exists of non-cancer fusions in humans as well.

Using the standard phylogenetic parsimony argument while considering both primate fusions and human fusions that have an NCBI gene ID, evidence exists for the independent recurrence of 6 gene pairs (Fig. 1). In 5 additional pairs, evidence exists for either the recurrence of fusions or other paths (Fig. 1). Taking into account also human fusions that lack an NCBI gene ID but are supported by other scientific reports (Table S2) shows many more cases of independent fusion recurrence (up to 38/132). Note that the pipeline was not intended to identify all separate genes in humans that are fused in primates, but rather to provide a small, validated sample of those.

## Discussion

Although it is known that genes fused in one organism often interact as separate genes in another (Marcotte et al., 1999; Enright et al., 1999; Enright & Ouzounis, 2001), this fact has been attributed to random mutation and natural selection–based causes (Marcotte et al., 1999). This attribution was subsequently criticized for invoking minute economic considerations (Doolittle, 1999), while leaving the reasons for the phenomenon unclear.

The used-fused hypothesis invokes mutational considerations instead (Livnat, 2017): it argues that genes that are used together are more likely to be fused together by mutational mechanisms (Livnat, 2017).

The tests above provide a set of results that bear on this topic: (i) The more tightly genes interact in one species where they are separate, the more likely they are to be found fused in other species, controlling for the genomic distance between pair members. (ii) This effect holds separately both for genes that are nearby and for genes that are distant from each other. (iii) Among genes that are separate in one species, those that are nearby each other are more likely to be fused in other species compared to those that are distant from each other. (iv) The more frequently genes that are separate in one species are observed in the same TAD, the more likely they are to be found fused in other species, controlling for the two-dimensional genomic distance between pair members. (v) The list of gene fusions in human cancers overlaps significantly with the list of gene fusions that occurred in evolution in other species. (vi) In the primate fusion dataset investigated, fusions predominate over fissions and often recur independently. We first argue that these facts favor the used-fused hypothesis over its alternatives (Table 1) and then discuss implications.

### Consideration of Alternative Hypotheses

Note that many of the pairs of genes that are fused in other organisms but separate in humans are nearby each other in humans (see for example Table 2 and Table S2). In and of itself, this observation could support $H_1$—the hypothesis that nearby genes are more likely than distant genes to interact with each other and are also more likely than other genes to undergo fusion mutation by random transcriptional read-through or deletion mutations unrelated to their interaction. However, this explanation is not sufficient on its own to account for the full range of observations, for several reasons. First, when looking at pairs of nearby genes of the same genomic distance between pair members, those pairs whose members interact more tightly are more likely to be found fused. Various measures of “working together” provide cross validation for this finding, including co-expression, co-localization, same-TAD presence and semantic similarity of GO terms (Tables 2, 3, 4, 5, S1).

Second, the random read-through or deletion mutation hypothesis does not directly account for the fact that the used-fused effect exists also in pair members that are distant from each other, whether in the same or in different chromosomes (Tables 2, 4, and 5). Although one could hypothetically assume that all fused genes were neighbors at the moment of their fusion in the species where they fused, that would be a restrictive assumption: the motivational *TRIMCyp* case described above is just one example from previous literature of fusion of non-neighbors, and the overlap between the evolutionary and cancer fusions includes several gene pairs whose members are distant from each other in humans yet become fused from a distance in human cancers.

Third, the finding that genes more commonly observed in the same TAD are more likely to undergo a fusion mutation while controlling for distance between pair members cannot be well explained by random read-through or deletion mutations.

$H_2$, according to which genes become fused by random mutation, and that among the fusions thus generated, those made by genes that had already been interacting prior to fusion are more likely to be favored by selection, covers any type of random mutation, including but not limited to read-through and deletion mutations fusing nearby genes. Thus, in principle, it could account for the increased tendency to observe fusions of pair members that interact more tightly with each other, whether they are nearby or distant from each other, though it predicts a different mechanism for how those came to be in the first place.

However, this hypothesis does not account for the cancer-overlap result—the result that genes that became fused in other organisms in the course of evolution are more likely than random pairs to become fused in human cancers (Table 6). Selection in non-human organisms favors mutations that increase the ability to survive and reproduce at the organismal level (e.g., improve foraging abilities, reduce predation risks, etc.), whereas selection among human cancer cells favors mutations that increase the ability of the cell to proliferate as a cancerous cell and its probability of being observed in tumors. The latter ability is not expected to match the former systematically and often comes at the expense of the former. This contrast between the selection pressures involved leaves ill-explained the cancer-overlap results under the purely selection-based explanation $H_2$.

In contrast, all of the findings are consistent with the hypothesis that genes that are used together are more likely to undergo a fusion mutation for mechanistic reasons inherent to their interaction (Livnat & Papadimitriou, 2016; Livnat, 2017). First, since nearby genes are more likely to be working together than remote genes (Michalak, 2008; Koonin, 2009; Ghanbarian & Hurst, 2015; Lian et al., 2018), this hypothesis is immediately consistent with the fact that many fusions occur between nearby genes. Second, unlike $H_1$, which argues that fusions arise by random read-through or deletion mutations, the used-fused hypothesis immediately accounts for the findings that (a) among same-distance nearby genes, those that interact more tightly are more likely to become fused (Tables 2, 4, 3, 5, S1); (b) among same-distance remote genes, those that interact more tightly are more likely to become fused (Tables 2, 4, 5, S1); and (c) the more frequently genes are observed in the same TAD, the more likely they are to become fused (Table 3). Third, unlike $H_2$, the used-fused hypothesis accounts for the propensity of the same gene pairs to undergo fusion in both evolution and cancer without hindrance: the likelihood of a fusion mutation is determined by mutational mechanistic phenomena, and the resulting fusion mutations could then undergo different selection pressures in each case, leaving a small but statistically significant overlap between evolutionary and cancer fusions, as observed. In other words, mutational mechanisms are a primary factor limiting the set of gene pairs with fusion potential, explaining the cancer-evolution overlap. The mechanistic explanation proposed here also explains the recurrence of fusions such as *TRIMCyp* observed in previous work (Virgen et al., 2008; Nisole et al., 2004; Sayah et al., 2004).

In addition to accounting for the evolution-cancer fusions overlap, the used-fused hypothesis also offers a more parsimonious explanation than $H_2$ for the finding that many but not all fusion-related genes are nearby each other. To explain these results without the used-fused hypothesis, one would have to invoke $H_2$ first to explain the finding that genes that interact more tightly are more likely to become fused also when distant from each other. However, it is problematic to use $H_2$ to account for the fact that many fusion-related genes are nearby each other: that would ignore the obvious potential of such genes to become fused more often than others for mutational reasons, even if such mutational reasons are limited to random read-through or deletion mutations and their ability to fuse specifically nearby genes. At the same time, using $H_2$ to account for the fusions of distant genes and adding $H_1$ to account for the fact that many fusion-related genes are nearby each other would now require using two different hypotheses, not only to address the effect in pairs of different distance categories, but even to address different findings involving pairs of the same distance category (pairs of nearby vs. distant genes and pairs of nearby genes with stronger vs. weaker interactions). Thus, adding up different hypotheses based on random mutation is a less parsimonious approach than using just the used-fused hypothesis, and even then addresses only a limited part of the results (it does not address well the cancer-overlap results).

**Fig. 2:**
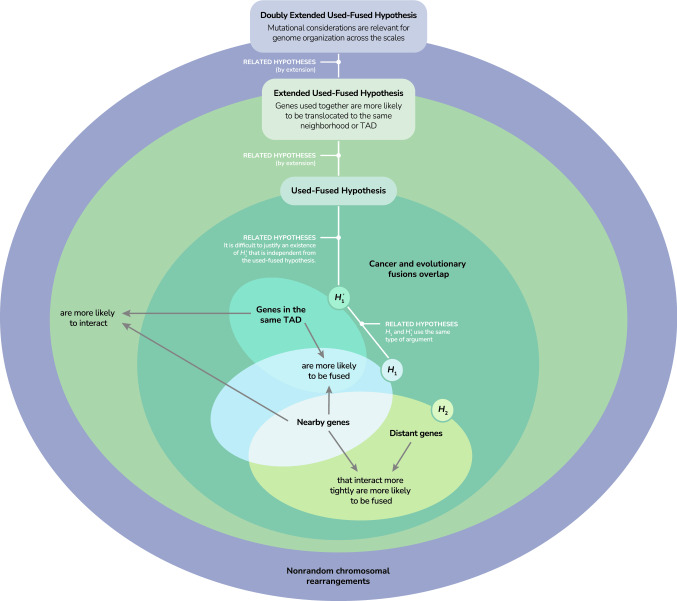
The used-fused hypothesis provides a parsimonious explanation for the findings. Findings of the present work (nearby genes are more likely to become fused than remote genes; nearby genes that interact more tightly are more likely to become fused than other nearby genes; distant genes that interact more tightly are more likely to become fused than other distant genes; genes more frequently observed in the same TAD are more likely to become fused, controlling for the 2D genomic distance between genes; evolutionary and cancer fusions significantly overlap) and previously known facts (nearby genes as well as genes in the same TAD are more likely to interact than other genes) are presented and grouped together by the hypotheses that could explain them. $H_1$: Random transcriptional read-through or deletion mutations underlie fusions between nearby genes in a manner not causally related to the fact that they are more likely than other genes to interact. $H^{\prime } _1$: Due to their proximity in 3D but unrelated to the fact that they work together, genes in the same TAD are more likely to undergo a fusion mutation. $H_2$: Genes that interact, once fused by a random mutation, generate a fusion that is more likely to be favored by selection compared to a fusion gene generated by random mutation from genes that do not interact. The used-fused hypothesis: genes that are used together are more likely to undergo a fusion mutation for mechanistic reasons involving their interaction. The extended used-fused hypothesis: genes that are used together are also more likely to be translocated to the same neighborhood or TAD for mechanistic reasons involving their interaction. The doubly-extended hypothesis: various mutational mechanistic considerations are relevant to the evolution of genome organization across the scales. As the diagram shows, $H_2$ does not explain the cancer-evolutionary fusions overlap and does not provide a parsimonious explanation for the other facts explained by the used-fused hypothesis because, on the one hand, it does not involve the obvious explanatory potential of mutational considerations in the cases of nearby genes and genes in the same TAD, and, on the other hand, invoking $H_1$, $H^{\prime } _1$ and $H_2$ together is unparsimonious, as the used-fused hypothesis accounts for all of the relevant facts in one. While $H_1$ and $H^{\prime } _1$ are related because they use a similar argument, it is hard to justify $H^{\prime } _1$ vis a vis used-fused hypothesis, since the mechanisms involved in the fact that genes in the same TAD work together are also expected to be involved in their probability of undergoing a fusion mutation. Thus, the facts explained by $H_1$ and $H^{\prime } _1$ are easily accounted for by the used-fused hypothesis. As a consequence, $H_1$ can be revised as a particular case of the used-fused hypothesis that involves mutational mechanistic considerations. The used-fused hypothesis not only replaces these other hypotheses, but is also extendable in principle to cover the fact that nearby genes and genes in the same TAD are more likely to interact together than other genes in the first place. More generally, mutational considerations could hypothetically contribute to chromosomal rearrangement hotspots (Pevzner & Tesler, 2003; Alekseyev & Pevzner, 2007) and thus be involved in genome organization evolution across the scales

Adding the TAD results further encumbers interpretations other than the used-fused one. The application of $H_2$ to these results is unsatisfying in the same way as its application to nearby genes. However, unlike $H_1$, it is difficult to argue here that ($H_1'$:) genes in the same TAD are more likely to undergo fusion mutation due to their proximity in 3D but unrelated to the fact that they work together, because the mechanisms of 3D proximity are inherently connected to the mechanisms of gene coexpression (Le Dily et al., 2014; Neems et al., 2016; Tarbier et al., 2020). According to the used-fused hypothesis, the same mechanisms due to which being in 3D proximity facilitates genetic interaction are also expected to facilitate fusion mutations (genes that work together are more likely to be expressed and thus have their chromatin open at the same time and place in the nucleus, allowing for various downstream mechanisms, whether retroposition, *trans*-splicing, recombination or more to increase the chance of fusion; Livnat and Papadimitriou, 2016; Livnat, 2017). Thus, to explain the co-expression, same-TAD presence, co-localization and GO term results without the used-fused hypothesis, multiple different hypotheses would be required, when the used-fused hypothesis accounts for all of these findings and more under one umbrella (Fig. 2).

### The Used-Fused Hypothesis and the Evolution of Genome Organization

The fact that the used-fused hypothesis explains the TADs result better than $H_1^{\prime }$ and the similarity between $H_1^{\prime }$ and $H_1$ allows us to rethink $H_1$—the proposal that nearby genes are simply fused by local read-through or deletion mutations and are also independently more likely to be working together. While it may appear easy to attribute these local mutations to the notion of random mutation, they actually involve at least a minimally mechanistic consideration: it is the genome architecture that determines which genes such mutations connect in the first place. Because the invocation of a mechanism here (the role of proximity between genes) appears minimal, it could be seen as fitting with the random mutation view when considered in isolation from other observations. However, the other findings obtained here raise the possibility that the used-fused mutational mechanistic framework explains gene fusions better than otherwise; that the fusion of neighbors may involve additional mutational mechanisms besides read-through and deletion mutations (for example, successful alternative splicing has to follow these mutations, which has been taken for granted until now but needs to be considered); and that fusion by read-through or deletion mutations itself may be seen as an example of the used-fused framework for involving genomic architectural considerations.

Indeed, we argue that from the beginning it is better to see the used-fused effect as an example of a broader phenomenon, where genes that are used together are more likely than others not only to undergo a fusion mutation but also to be moved by a translocation mutation into the same neighborhood when initially distant, as the same sorts of mutational mechanisms proposed for the fusion case would apply also to translocations. In fact, the observation that nearby genes are more likely to be fused than distant ones suggests that usually genes that interact remotely first move to the same neighborhood and later in evolution become fused. Indeed, it has been proposed that much evolutionary time may elapse between the steps of interacting from afar, translocating to the same neighborhood and fusing (Livnat, 2013, 2017).

This extension of the used-fused hypothesis offers an explanation for why neighboring genes, or genes in the same TAD, are more likely than other genes to be working together in the first place. Indeed, these facts are in need of an explanation because we know that the genome is substantially reorganized over long periods of evolutionary time (Graur & Li, 2000), yet genes that work together are more likely to be found in the same neighborhood, raising the possibility that active movement to the same neighborhood, as opposed to the mere evolution of new interactions between sedentary neighboring genes, factors into the reorganization. Absent a mutational explanation such as this, one has to either accept that it just so happens that these facts of genome organization exist, or invoke arguments based on random mutation and natural selection, such as that selection will favor the moving to the same neighborhood or the fusion of genes that work together because this will save energy or time or avoid errors in the process of their expression—arguments of a sort that has been questioned for involving minute economic considerations (Doolittle, 1999). In contrast, both the phenomena of gene fusion studied here and the phenomenon of neighborhoods of genes that work together can be accounted for by the extended used-fused hypothesis under one umbrella and without resorting to such considerations. This view furthermore fits better with evidence for the fact that breakpoints of chromosomal rearrangements due to reversals, translocations, fissions and fusions occur in hotspots (Pevzner & Tesler, 2003; Alekseyev & Pevzner, 2007) as opposed to being randomly distributed across the genome (Nadeau & Taylor, 1984; Sankoff & Trinh, 2005), opening up the possibility that mutational considerations are relevant for the evolution of genome organization across the scales (Fig. 2).

With this expanded understanding, we can now rethink both $H_1$ and $H_2$. $H_1$ speaks of distance alone and $H_2$ speaks of interaction alone, both from the perspective of random mutation and natural selection. The evidence suggests that each provides a limited and inaccurate picture of reality. We argue that, in reality, fusion and translocation mutations are due to the expanded used-fused effect, where interaction and distance are inseparable. Interaction itself determines the evolution of genome organization and hence distance in the long term, and both interaction and distance affect fusion probability through mutational mechanisms.

### Fusion vs. Fission

Had the data represented overwhelmingly fission rather than fusion events, it could not have been used to support the used-fused hypothesis. However, this possibility seems unlikely, as it can be countered in three different ways. First, in the case of cancer, the events considered are fusions, not fissions. It seems to be an unlikely assumption that the gene pairs that repeat in both cancer and evolution are fusion events in cancer but fission events in evolution. More likely, they are fusion events in both, enabling a parsimonious explanation via mutational mechanisms of the cancer-evolution fusions overlap. Second, previous literature does not favor fission over fusion inference overall (Kummerfeld & Teichmann, 2005). Third, and consistent with these points, we found that fusions dominate fissions in the primate fusion data ${\sim }50:1$. Together, these results make it unlikely that historical fission events change the conclusion with regards to the causes of fusion inferred here.

### Phylogenetic Recurrence of Fusions

The multiple cases of fusion recurrence revealed by the phylogenetic analysis of the primate data further supports the used-fused hypothesis. Under the used-fused effect, the fact that related species share many of the same genetic interactions is expected to lead to recurrence of fusions. In fact, the argument is even stronger: Under the used-fused framework, the standard phylogenetic parsimony method may severely underestimate the ratio of fusions to fissions and the extent of fusion recurrence (Fig. 1) because the similarity between related species is expected to increase the probability of parallel fusion that will go undetected by this method. Therefore, cases that appear under the standard parsimony method as cases of fission recurrence or of a single-fusion origin may actually be cases of extensive parallel fusion. This increases the probability that some or all of the unresolved histories of Fig. 1 are cases of fusion rather than fission recurrence.

### The Used-Fused Hypothesis Applies to Both Germ and Soma

If the somatic expression of genes that work together in the soma is not reflected in some manner in their expression in the germ cells, the used-fused hypothesis would be limited to pairs of genes that serve germline functions. We made two advances toward studying this requirement. First, because we focused on humans, primates and other multicellular organisms appearing in STRING, evidence for the used-fused effect in these organisms in gene pairs not selected by tissue serves as *prima facie* evidence that the used-fused effect applies also to pairs of genes that interact in the service of somatic functions. Second, we further tested this possibility by actively excluding gene expression data from the germline tissues and obtained the used-fused effect even with this exclusion. This fact further supports the hypothesis that the used-fused effect applies also to gene pairs whose interactions serve somatic functions, potentially implicating the germline phenomenon of transcriptional promiscuity in gene fusion, as originally proposed (Livnat, 2013; Livnat & Papadimitriou, 2016; Livnat, 2017). Future research will be needed to explore the question of the degree to which genes that serve somatic functions undergo the used-fused effect and the molecular biological mechanisms that enable them to do so. Connected to this topic, we discuss further predictions regarding transcriptional promiscuity in a subsequent section below.

### Implications

#### Mutational Mechanisms and the Evolution of Genome Organization

The used-fused hypothesis offers to account for both parallel gene fusions in evolution (Carvalho et al., 2010; Livnat, 2013) and recurrent gene fusion in genetic disease and cancer (Li et al., 2008; Osborne, 2014) ﻿under one umbrella. Extending this line of thinking, we further hypothesized here that mutational mechanisms are relevant not only to fusion but also to translocation of interacting genes into the same neighborhood (Michalak, 2008; Koonin, 2009; Ghanbarian & Hurst, 2015; Lian et al., 2018). Both the used–fused hypothesis and its extension avoid the problem of minute economic considerations and offer a broad parsimonious view according to which the evolution of genome organization is driven to a large extent not by random mutation and random genetic drift (cf. Lynch 2007) but by mutational mechanisms.

#### Mutational Mechanisms and Exon Shuffling

Exon shuffling—where exons from different genes join together in new combinations in the course of evolution, creating new genes—is a phenomenon in evolutionary time. Alternative splicing—where mRNAs from different exons come together in different combinations to create multiple alternative protein products—is a phenomenon in developmental time. Therefore, by showing that gene pieces are copied and translocated for mutational reasons between genes that interact, our data makes a concrete connection, via a mutational mechanism, between evolution and development: it demonstrates that selection acting on phenomena in developmental time affects the interactions between exons over the generations and leaves an imprint in the genome that in turn affects the probabilities of mutation origination. Thus, developmental phenomena affect the probabilities of mutation origination.

To expand, from the random mutation perspective, Gilbert’s famous exon-shuffling hypothesis (Gilbert, 1978) implies that the intron-exon structure of eukaryotic genes facilitates the generation of new genes in evolution by allowing presumably random rearrangement breakpoints to fall outside of exons and thus avoid disrupting coding regions (Gilbert, 1978). However, our results raise the possibility that exon shuffling is not simply the result of random mutation: exons first interact from afar, and their interaction leads them via mutational mechanisms to be translocated to interact in *cis* and to become fused (Livnat, 2017). This contrast between random mutation and the used-fused hypothesis is particularly clear in cases where the same exons are *trans*-spliced in one species or population and *cis*-spliced in another, as is the case of the exons of the eri-6 and eri-7 genes in *C. elegans* strain N2 and the corresponding exons of the fused homologs in *C. briggsae* (Fischer et al., 2008), and in cases where some functions, such as the production of fatty acids from acetyl-CoA, are achieved by multiple single-module proteins in one taxon but by a single multi-module protein in another (Graur & Li, 2000). Such cases are easier to understand based on the used-fused hypothesis (Livnat, 2013, 2017) than from the random-mutation–based view of exon shuffling. Indeed, under the used-fused hypothesis, the intron-exon structure facilitates exon shuffling in a far deeper and more powerful way than allowed under random mutation, as phenomena of the alternative splicing machinery could contribute to exon shuffling through mutational mechanisms (e.g., *trans*-splicing being replaced by *cis*-splicing or by fusion at the DNA level mutationally via used-fused mechanisms).

#### Mutational Mechanisms and the Fitness Distribution of Mutations

It is often said that because most observed mutational effects are detrimental, mutation must be random (Fisher, 1930). However, as follows from Livnat (2013, 2017), Livnat and Papadimitriou (2016), Melamed et al. (2022) and Livnat and Melamed (2022), the fitness distribution of mutations could have leaned more to the detrimental side than it does in reality if fusion mutations were unrelated to the organism’s structure and function. Indeed, in accord with the notion of random mutation, it was originally proposed that detrimental mutations should be more common than beneficial ones (Fisher, 1930), and only later was it discovered that the vast majority of substitutions appear neutral or nearly so (Kimura, 1968; King & Jukes, 1969). That finding was then explained in various ways, including that synonymous mutations have no effect (King & Jukes, 1969), that the majority of the genome is non-functional and thus the majority of mutations are of no effect (Ohno, 1972), and that the majority of the genome consists of regulatory regions where mutations often have little effect (King & Wilson, 1975; Ohta, 2002). However, not mutually exclusive with these possibilities, our results raise the possibility that the fitness distribution of mutations is also affected by mutational mechanisms: if genes that are used together are more likely to undergo a fusion mutation, gene fusions are less accidental than conceptualized under the notion of random mutation and may be less disruptive or more beneficial compared to random gene fusions. Indeed, the used-fused effect ties the specific causes of a mutation to its potential consequences. Recent studies extend to other mutation types the possibilities that mutational mechanisms affect the fitness distribution of mutations and that the specific causes of a mutation are related to its consequences (Livnat, 2017; Melamed et al., 2022; Livnat & Melamed, 2022).

#### Evolutionary Parallelism

It has been suggested that parallel mutations are more likely to be observed in more closely related species because they experience more similar selection pressures and have a more similar genetic and developmental background on which the phenotypic consequences of random mutations depend (Blount et al., 2018; Ord & Summers, 2015). However, if mutational mechanisms that are affected by genetic and epigenetic information that is present in the germline genome influence the probabilities of specific mutations as exemplified by the used-fused effect, then mutational mechanisms constitute another reason for the increased parallelism between related species.

Other recent empirical findings connecting mutational phenomena to parallelism in general and adaptive evolution specifically include the findings that the human hemoglobin S mutation, which protects against malaria in heterozygotes and causes sickle-cell anemia in homozygotes, originates significantly more rapidly than expected by chance for this mutation type, specifically in Africans (Melamed et al., 2022), and that high rates of deletion of a specific enhancer are responsible for the parallel and likely adaptive loss of the pelvic hindfin in freshwater sticklebacks (Xie et al., 2019) (see more in Kratochwil et al., 2019; Kratochwil and Meyer, 2019; Lind, 2019). Henceforth the possibility of extensive parallelism due to mutational mechanisms must be taken into account in the interpretation of phylogenetic and experimental evolution data.

Given the used-fused effect, even if many or most of the species in a clade share a certain gene fusion, it could be that their common ancestor shared the tendency to generate the fusion, and that the fusion arose later independently multiple times. This new interpretation offers to resolve a contradiction in previous data, where authors examining distant species concluded that fusions are more common than fissions (Snel et al., 2000; Kummerfeld & Teichmann, 2005), whereas authors examining more closely related species concluded the opposite (Leonard & Richards, 2012). It is possible that fusions are always more common than fissions but show extensive parallelism in closely related species, which then appears as fission when interpreted according to the standard phylogenetic parsimony argument.

#### Mutational Activity in the Germline and Cancer

One consequence of the used-fused hypothesis is that genetic activity in the germline must reflect that of the soma in some respect (Livnat, 2013, 2017). It has been proposed that the germline-specific phenomenon of transcriptional promiscuity (TP) underlies this correspondence (Livnat, 2013, 2017). However, if all somatic genes were active in the germline at the same time, that would not have allowed specifically pair members that are used together in the soma to find each other in the germline. Combining this observation and the used-fused hypothesis therefore implies that there must be further structure in TP. This leads us to predict that TP consists of waves of gene activation in the germ cells that expose close connections in the somatic regulatory genetic networks without betraying the full coordinated activity of any whole somatic network (the latter could not have taken place in the germline).

This prediction is surprising because it implicates much molecular machinery in the evolutionary process. However, it furthermore connects to the overlap found here between cancer and evolutionary fusions. Based on their observations that cancer cells imitate germ cells and trophoblasts in many respects, including global hypomethylation, expression of chorionic gonadotropin, downregulation of the major histocompatibility complex, the power of proliferation, the expression of cancer/testis (CT) antigens, and more, Old and collaborators proposed that cancer cells activate a gametogenic program (Old, 2001; Simpson et al., 2005). Combining this point with the importance of mutation-affecting phenomena in the germ cells including TP (Livnat, 2013) suggests that cancer cells could be sharing to some degree the mutational activity that normally takes place in the germ cells (Livnat, 2013). Together with the used-fused effect, this provides a concrete mutational connection between cancer cells and germ cells and offers a unified account for the otherwise highly disparate facts that the used-fused effect applies to somatic genes and that an overlap exists between cancer and evolutionary fusions.

This connection between germ cells, cancer and the used-fused effect raises the meaning of cancer as an evolutionary disease to the next level: so far, the analogy between cancer and evolution implied that cancer cells evolve within the individual by random mutation and natural selection acting on these cells’ ability for cancerous proliferation. However, the above suggests that cancer may be an evolutionary disease not only in the sense that it can change under selection, but also in the sense that it shares mutational mechanisms with the process of evolution. This may offer new ways of thinking about cancer. Indeed, the mutations occurring in cancer in a certain species or population at a certain time may reflect to some degree the mutations occurring naturally in the evolution of that ﻿species or population at that time.

#### Mutational Chunking of Pieces of Information in Molecular Evolution

An important question is the meaning of the used-fused effect for evolution. Consider the phenomenon of gene duplication via mutational mechanisms such as non-allelic homologous recombination, non-homologous end-joining, retroposition and other mechanisms (Lupski, 1998; Gu et al., 2008; Woodward et al., 2005; Lee et al., 2006; Hastings et al., 2009; Korbel et al., 2007; Lupski, 2006). It would be difficult to argue that such mutational mechanisms evolved under random mutation and natural selection for the reason that they allowed for gene duplication: such a benefit is a long-term one, whereas random mutation and natural selection is typically based on individual-level, immediate benefits (Williams, 1966; Dawkins, 1976). At the same time, evolution as we know it would not have been possible without the existence of these duplication mechanisms, as gene duplication is of fundamental importance to evolution (Ohno, 1970; Jacob, 1977). Likewise, it is of interest to note that the chunking of pieces of information that are repeatedly used together into a single unit is a powerful principle across different processes of information acquisition (Hebb, 1949; Lindley, 1966; Löwel & Singer, 1992; Tulving & Craik, 2005) and that evolution has been thought of as a process of information acquisition, where genetic information is acquired under natural selection (Livnat, 2013, 2017). As in the case of gene duplication, noting this potential benefit of the fusion of genes that work together is not to say that the used-fused effect itself evolved by random mutation and natural selection based on this benefit, but rather to recognize that it is an interesting and potentially important property of the genetic system as a whole, whose own origin, as the origin of the phenomenon of gene duplication, requires its own investigation.

Indeed, much information is involved in determining the interactions between two genes, including but not limited to transcription factor binding sites, epigenetic modifications and chromatin states at the interacting loci and at other loci regulating them. Therefore, when genes that have evolved to interact tightly become fused in the course of evolution, simplification of gene regulation results: what previously required two separate arms of regulation now requires one. Thus, the local outcome of the used-fused effect is simplification of preexisting genetic interactions and their replacement by a new genetic state.

Recent work has suggested that the used-fused effect shares the principles of mutational replacement and simplification with other mutation types: that genes expressed above their norm as a result of an evolutionary response to environmental change are more likely to undergo a gene duplication mutation via elevated-transcription–based gene duplication mechanisms (Livnat, 2017; Melamed et al., 2022; Livnat & Melamed, 2022); that RNA editing of a given nucleotide may lead to the corresponding DNA change via RNA-editing–based mutational mechanisms (Melamed et al., 2022; Livnat & Melamed, 2022); and more (Melamed et al., 2022; Livnat & Melamed, 2022). It has been proposed that the interaction between mutational mechanisms and selection over the generations involves a process of simplification under performance pressure (Livnat, 2017).

## Conclusions

The fact that, considering genes that are separate in one species, the more tightly they interact the more likely they are to be found fused in other species; the fact that this applies separately both to genes that are nearby and to genes that are distant from each other; the fact that genes that are nearby each other are more likely to be found fused in other species compared to genes that are remote from each other; the fact that the more frequently genes that are separate in one species are observed in the same TAD, the more likely they are to be found fused in other species; the fact that the list of gene fusions in human cancers overlaps significantly with the list of evolutionary gene fusions in other species; and the facts that fusions predominate over fissions and sometimes recur independently all favor the hypothesis that genes that are used together are fused together more than others for mutational mechanistic reasons related to their interaction. This outcome has multiple implications. First, it offers a unifying explanation for the recurrence of gene fusions both in evolution and in genetic disease and cancer, and avoids the need to invoke minute economic considerations or pure chance in explaining the empirical patterns. Second, it implies that exon shuffling is facilitated by mutational mechanisms involving genetic interactions and that the intron–exon structure may play a role in exon shuffling deeper than the random-mutation view implies. Third, it raises the possibility that fusion mutations are less detrimental or more beneficial in reality compared to what they could have been if they occurred purely at random, thus demonstrating that mutational mechanisms can contribute to the fitness distribution of mutations. Fourth, it demonstrates that mutational mechanisms could contribute to evolutionary parallelism and raises the possibility of extensive parallelism in fusion mutations, with implications for the interpretation of phylogenetic evidence. Fifth, it suggests that transcriptional promiscuity and/or other germline-specific phenomena may be involved in evolutionarily relevant mutational mechanisms, that cancer and germ cells may share mutational mechanisms to some degree, and that cancer may be considered an “evolutionary disease” not only because cancer cells may be seen as undergoing selection but also for mutational reasons. Sixth, the fact that the fusion of pieces of information that are repeatedly used together is useful in other processes of information acquisition does not imply that the used-fused effect evolved under random mutation and natural selection for that purpose, though it makes it possible that the used-fused effect is important for evolution, much as is the case for gene duplication. Seventh, multiple types of mutation may represent local replacement and simplification of preexisting genetic interactions. Finally, we hypothesized here that genes that are used together are also more likely to be translocated to the same neighborhood or TAD for mutational mechanistic reasons, and that mutational mechanisms, as opposed to random mutation and random genetic drift, are important for the evolution of genome organization across the scales. Future research is needed to explore all of these consequences in detail.

## Supplementary Information

Below is the link to the electronic supplementary material.

Supplementary file1 (PDF 687 kb)

## Data Availability

The datasets analysed during the current study are referenced in the text and publicly available.
