# Supplementary material for: Genes that are Used Together are More Likely to be Fused Together in Evolution by Mutational Mechanisms: A Bioinformatic Test of the Used-Fused Hypothesis
Source: Evol Biol. 2022 Nov 30;50(1):30–55. doi: 10.1007/s11692-022-09579-9 (PMC9925542; doi:10.1007/s11692-022-09579-9)
Supplement: Supplementary file 1 — Supplementary file1 (PDF 687 kb) [file 11692_2022_9579_MOESM1_ESM.pdf]

Supplemental Information

Genes that are used together are more likely to be fused together in evolution by mutational mechanisms: A bioinformatic test of the used-fused hypothesis

Evgeni Bolotin, Daniel Melamed, Adi Livnat*

* To whom correspondence should be addressed; e-mail: alivnat@univ.haifa.ac.il

This PDF file includes the below:

Supplemental Text S1 to S4

Supplemental Tables S1, S2, and S3

S1. Further information regarding distance groups

While classifying gene pairs into distance groups, in cases where the start position of the downstream gene was upstream to the end position of the upstream gene, we considered the genes "overlapping." In cases where the entirety of one gene was included in the interval of the other gene, we considered the genes "included." Gene pairs in the "overlapping" or "included" categories, as well as gene pairs with no protein-coding genes between the pair members, were all considered neighbors (SC_0). If one of the genes in a pair was on a non-localized/unplaced scaffold or on the alternate loci assembly, the distance between these two genes was considered unclear and the gene pair was excluded from further analyses.

S2. Determining the alignment length threshold for the primate fusions analysis

All features in the human genome found in the primary genome assembly and marked as an exon of a protein-coding gene were extracted from the human 'genomic.gff' file (downloaded from the NCBI repository; NCBI, 2004) and the lengths of all of the exons were measured. Given that exons are basic units of proteins and that >90% of human exons were of a length equal to or longer than 20 amino acids, providing sufficient coverage while avoiding alignments that are too short and are likely to generate random-matches, we chose 20 amino acids as the alignment length threshold.

S3. Determining the alignment identity threshold for the primate fusions analysis

To find protein fusions, one needs to obtain an estimate of the expected identity between homologous protein-coding regions, which differs between pairs of species. Therefore, for each primate species, we compared the human and that primate's protein datasets using FASTA (Pearson and Lipman, 1988) to identify pairs of bi-directional best-hit (BBH) proteins (where each protein has the other as its best hit) and, consistent with previous literature, considered BBH protein pairs that had at least 30% identity over an aligned region whose length was at least 70% of the shortest protein length as putative homologs (Konstantinidis and Tiedje, 2005; Rost, 1999). Based on histograms of alignment identity percents binned into 10% groups, we chose 60%, 70%, 70%, 80%, 90% and 90% as the minimum identity percent cutoff for Mouse lemur,

Common marmoset, Owl monkey, Pig-tailed macaque, Gorilla and Chimpanzee, respectively, thus covering $\sim$99% of putative homologs in each case.

S4. Similarity analysis for the identification of likely human homologs of primate fused proteins

All of the candidate human proteins were divided into groups according to the region of the primate protein to which they were aligned and, from each group, the best alignment, defined as the alignment that had the highest identity percent, was used. Fused primate proteins for which likely human homologs were not identified due to the fact that proteins from multiple human genes aligned to them with similar quality were excluded from further analyses.

To cross-validate the above method for finding homology, we checked for a similarity in gene names between any of the best aligned human genes and the fused primate genes, because gene names in newly sequenced species are often assigned based on similarities to genes already known in other species. In addition we checked for synteny between the genomic regions flanking the primate gene and those flanking the human genes aligned to it, even though by definition perfect synteny cannot be obtained for fusions. In many cases, name similarity or synteny or both appeared, strengthening our confidence in the list of fusions.

To analyze synteny, we extracted the 50,000 bp-long upstream and downstream regions of each primate gene and all human genes aligned to it. The boundaries of the genes were identified using the gene feature table of each species downloaded from the NCBI repository (NCBI, 2004). If the length of the upstream or the downstream region in any of the compared genes was shorter than 50,000 bp, that actual length was used for the analysis in all aligned genes for uniformity. Upstream or downstream regions shorter than 10,000 bps were considered too short for the synteny analysis, and the given region was excluded from analysis in all compared genes. The regions flanking the human genes were compared to the regions flanking the primate gene in a pairwise manner using the program mySyntenyPortal (Lee *et al.*, 2018). The synteny strength was determined based on the length of the synteny blocks identified between the compared flanking regions. Upstream and downstream regions were analyzed separately.

References

Konstantinidis, K. T. and Tiedje, J. M. (2005). Towards a genome-based taxonomy for prokaryotes. *Journal of Bacteriology*, *187*(18), 6258–6264

Lee, J., Lee, D., Sim, M., Kwon, D., Kim, J., Ko, Y., and Kim, J. (2018). mySyntenyPortal: an application package to construct websites for synteny block analysis. *BMC Bioinformatics*, *19*(1), 216

National Library of Medicine (US), National Center for Biotechnology Information (2004). NCBI RefSeq. Retrieved Oct 2018 from https://www.ncbi.nlm.nih.gov/refseq/.

Pearson, W. R. and Lipman, D. J. (1988). Improved tools for biological sequence comparison. *Proceedings of the National Academy of Sciences USA*, *85*(8), 2444–2448

Rost, B. (1999). Twilight zone of protein sequence alignments. *Protein Engineering*, *12*(2), 85–94

Table S1. *P-values* of co-expression comparison between fusion-related and control gene pairs using GTEx database and calculating mean co-expression value across tissues.

		Genomic control			String control		
	Distance[a]	Group size[b]	*p-value*[c]	W[c]	Group size[b]	*p-value*[c]	W[c]
**Mean co-expression of all tissues**	All pairs	9423-94230	<2.20E-16	5.74E+08	9423-93514	<2.20E-16	4.72E+08
	Same chromosome	1813-18130	<2.20E-16	1.97E+07	1813-17414	1.33E-05	1.67E+07
	SC_0	752-7520	<2.20E-16	3.40E+06	752-6817	1.37E-08	2.88E+06
	SC_1-99	652-6520	6.20E-08	2.39E+06	652-6520	7.75E-01	2.09E+06
	SC_100-499	267-2670	<2.20E-16	4.65E+05	267-2670	3.41E-02	3.81E+05
	SC_500+	142-1420	7.25E-09	1.30E+05	142-1407	3.07E-01	1.02E+05
	Different chromosomes	7610-76100	<2.20E-16	3.81E+08	7610-76100	<2.20E-16	3.11E+08
		Genomic control			String control		
	Distance[a]	Group size[b]	*p-value*[c]	W[c]	Group size[b]	*p-value*[c]	W[c]
**Mean co-expression of all tissues excluding testis and ovaries**	All pairs	9423-94230	<2.20E-16	5.74E+08	9423-93514	<2.20E-16	4.72E+08
	Same chromosome	1813-18130	<2.20E-16	1.96E+07	1813-17414	1.65E-05	1.67E+07
	SC_0	752-7520	<2.20E-16	3.39E+06	752-6817	1.27E-08	2.88E+06
	SC_1-99	652-6520	1.70E-07	2.38E+06	652-6520	8.14E-01	2.08E+06
	SC_100-499	267-2670	<2.20E-16	4.66E+05	267-2670	2.78E-02	3.82E+05
	SC_500+	142-1420	1.17E-08	1.29E+05	142-1407	3.26E-01	1.02E+05
	Different chromosomes	7610-76100	<2.20E-16	3.81E+08	7610-76100	<2.20E-16	3.11E+08
		Genomic control			String control		
	Distance[a]	Group size[b]	*p-value*[c]	W[c]	Group size[b]	*p-value*[c]	W[c]
**Mean expression per tissue**	All pairs	9423-94230	<2.20E-16	5.35E+08	9423-93514	4.08E-09	4.56E+08
	Same chromosome	1813-18130	<2.20E-16	2.00E+07	1813-17414	3.83E-16	1.76E+07
	SC_0	752-7520	4.39E-09	3.19E+06	752-6817	7.39E-04	2.74E+06
	SC_1-99	652-6520	<2.20E-16	2.78E+06	652-6520	1.78E-15	2.52E+06
	SC_100-499	267-2670	4.07E-12	4.47E+05	267-2670	6.57E-03	3.89E+05
	SC_500+	142-1420	3.55E-06	1.24E+05	142-1407	2.39E-01	1.04E+05
	Different chromosomes	7610-76100	<2.20E-16	3.48E+08	7610-76100	7.27E-03	2.94E+08
		Genomic control			String control		
	Distance[a]	Group size[b]	*p-value*[c]	W[c]	Group size[b]	*p-value*[c]	W[c]
**Mean expression per tissue excluding testis and ovaries**	All pairs	9423-94230	<2.20E-16	5.35E+08	9423-93514	1.65E-08	4.56E+08
	Same chromosome	1813-18130	<2.20E-16	2.00E+07	1813-17414	1.04E-15	1.76E+07
	SC_0	752-7520	2.19E-09	3.19E+06	752-6817	4.44E-04	2.75E+06
	SC_1-99	652-6520	<2.20E-16	2.76E+06	652-6520	3.31E-14	2.50E+06
	SC_100-499	267-2670	1.15E-12	4.49E+05	267-2670	5.24E-03	3.90E+05
	SC_500+	142-1420	3.35E-06	1.24E+05	142-1407	2.50E-01	1.03E+05
	Different chromosomes	7610-76100	<2.20E-16	3.48E+08	7610-76100	1.26E-02	2.94E+08

a) Distance is measured by the number of protein coding genes separating genes in the analyzed pair
b) Number of co-expressed genes in the fusion-related (left) and control group (right). The control group represents a 10x larger group than the fusion-related group. If for a certain distance group the number of possible control pairs was smaller than 10x the number of fusion-related pairs, all available control pairs were used for the analysis.
c) One-sided Mann-Whitney test statistics

Table S2. Human genes whose homologs are fused in at least one of the six primate species under study

Human genes' names	Human genes' ID	Distance (gene number)	Distance (bp)	Primate gene IDs						Evidence for neighbor fusion	Evidence for non- neighbor fusion	Cancer-associated fusions	Supporting databases and references
				M. Murinus	*C. jacchus*	*A. nancymaae*	*M. nemestrina*	*G. gorilla*	*P. troglodytes*				
AKT2/C19orf47	208/126526	0	7212						456035	√			[10]
C1QA/C1QC	712/714	0	3935	105873631					749231	√			[1; 17]
GUSB/ASL	2990/435	0	93475			105704845				√		√	[11]
IFNAR2/IL10RB	3455/3588	0	1847		100409946					√		√	[18]
INSL3/JAK3	3640/3718	0	3208						748892	√			[14]
MAG/CD22	4099/933	0	15359						450167	√			[1]
MC1R/TUBB3	4157/10381	0	1032		100394345			101131674		√			[6]
RPS15A/ARL6IP1	6210/23204	0	1333		100409293					√		√	[11; 10]
UCHL3/LMO7	7347/4008	0	14414						452604	√		√	[13; 11; 10]
PIP5K1A/PSMD4	8394/5710	0	5164			105724243				√			[3]
FAM53B/EEF1AKMT2	9679/399818	0	13470	105880357						√			[2]
SRA1/APBB3	10011/10307	0	175	105862529						√			[12]
KCNK7/MAP3K11	10089/4296	0	1759		100387411					√			[7]
CLSTN1/CTNNBIP1	22883/56998	0	23750	105886064						√		√	[16; 11; 10]
RBM34/ARID4B	23029/51742	0	5279					101138018		√			[19]
RAD54L2/TEX264	23132/51368	0	2515				105480508			√			[17]
SH3BP1/PDXP	23616/57026	0	2611			105721773				√			[9]
PRKD3/QPCT	23683/25797	0	19825		100396713					√			[10]
ABHD14A/ACY1	25864/95	0	2084			105722879				√			NCBI Gene ID: 100526760 [4]
TRIM58/OR2W3	25893/343171	0	15449				105474740			√			[8]
EEF1AKMT3/TSFM	25895/10102	0	204		100389321					√		√	[11]
OTUD6B/LRRC69	51633/100130742	0	15524		100407751					√			[10]
CHMP3/RNF103	51652/7844	0	39896	105876619		105731242	105465405			√		√	NCBI Gene ID: 100526767 [4]; [11]
ELOVL1/MED8	64834/112950	0	15834	105877420						√		√	[15]
PIGZ/MELTF	80235/4241	0	32329				105470789			√		√	[11]
AARSD1/PTGES3L	80755/100885848	0	3590			105711178				√			NCBI Gene ID: 100885850 [4]
TMEM120A/STYXL1	83862/51657	0	1146					101137372		√		√	[22; 10]
DTX2/UPK3B*	113878/105375355	0	4347						107966274	√			NCBI Gene ID: 441263 [4]
WFDC6/EPPIN	140870/57119	0	1131						458284	√			NCBI Gene ID: 100526773 [4]
GIMAP1/GIMAP5	170575/55340	0	13068		103794953					√			NCBI Gene ID: 100527949 [4]
TBCEL/TECTA	219899/7007	0	11890						744604	√		√	[20; 5; 21]
CFAP53/MBD1	220136/4152	0	360			105709036				√		√	[18; 10]
LY6G6F/LY6G6D	259215/58530	0	4677				105497620			√			NCBI Gene ID: 110599563 [4]
CDRT4/TVP23C	284040/201158	0	34653					101149725		√			[10]
SCO2/TYMP	9997/1890	O	O				105489744			√		√	[23]
TRIM58/OR2T8	25893/343172	1	39058						469159		√		[8; 10]
DNAAF10/PPP3R1	116143/5534	1	21297					101154030			√	√	[11]
LGALS9C/LGALS9	654346/3965	44	9136203				105476929				√	√	[11]
CDRT4/TVP23B	284040/51030	50	3313384						742740		√		NCBI Gene ID: 100533496 [4]
PMS2/DTX2*	5395/113878	338	70452549						100611882		√		NCBI Gene ID: 441263 [4]
PMS2/UPK3B*	5395/105375355	339	70501236						100612891		√		NCBI Gene ID: 441263 [4]
CR2/CR1	1380/1378	0	6233						449643				
EFNB3/DNAH2	1949/146754	0	5979			105720743							
FGF5/CFAP299	2250/255119	0	30248						461218				
GANC/CAPN3	2595/825	0	5834					101139167					
PLIN1/PEX11A	5346/8800	0	2114	105870617									
TEAD3/TULP1	7005/7287	0	790	105861386									

*Part of DTX2P1-UPK3BP1-PMS2P11 readthrough pseudogene

Table S2. Continued

Human genes' names	Human genes' ID	Distance (gene number)	Distance (bp)	Primate gene IDs						Evidence for neighbor fusion	Evidence for non-neighbor fusion	Cancer-associated fusions	Supporting databases and references
				M. Murinus	*C. jacchus*	*A. nancymaae*	*M. nemestrina*	*G. gorilla*	*P. troglodytes*				
TNFRSF4/SDF4	7293/51150	0	96			105731883							
FCN3/CD164L2	8547/388611	0	4319		100390159	105708427							
MKNK1/MOB3C	8569/148932	0	3404	105877503									
NPM2/FGF17	10361/8822	0	2800				105482096						
CLEC10A/ASGR2	10462/433	0	21015			105720767							
HTATIP2/PRMT3	10553/10196	0	3747	105854903									
CBY1/TOMM22	25776/56993	0	8099					101129688					
TLR9/TWF2	54106/11344	0	2447	105875040	100411708	105722888	105480535	101154047	470827				
RASIP1/IZUMO1	54922/284359	0	55				105478655						
IL36G/IL36A	56300/27179	0	19789						738914				
ERGIC1/RPL26L1	57222/51121	0	992		100393371								
CELF6/HEXA	60677/3073	0	23251			105716563			748732				
IGFLR1/U2AF1L4	79713/199746	0	77			105707061							
TREML2/TREML4	79865/285852	0	27054		100394702								
MRPL53/CCDC142	116540/84865	0	17				105465298						
TAGAP/LOC112267968	117289/112267968	0	13444	105868620									
CYB5D1/CHD3	124637/1107	0	18678				105473767						
C5orf24/TXNDC15	134553/79770	0	14035			105707263							
ASB14/DNAH12	142686/201625	0	651					101125647					
FAM53A/SLBP	152877/7884	0	8418	105881641									
ANKS6/GALNT12	203286/79695	0	11157					101127419					
CFAP299/BMP3	255119/651	0	67022	105880970									
ERICH2/GAD1	285141/2571	0	14239			105728239							
JMJD8/WDR24	339123/84219	0	176		100402756								
METTL2A/TLK2	339175/11011	0	20815						107971619				
KCNJ1/C11orf45	3758/219833	1	32192				105476213						
CPNE1/NFS1	8904/9054	1	3732		100389040								
SPIRE1/CEP76	56907/79959	1	142		100411109								
PCNX4/PPM1A	64430/5494	1	110938			105724084							
C11orf1/HSPB2	64776/3316	1	28653			105729625							
SARNP/DNAJC14	84324/85406	1	3204						467031				
RNFT2/FBXW8	84900/26259	1	57318			105728572							
NOP9/LTB4R2	161424/56413	1	1025				105477869						
LRRC43/B3GNT4	254050/79369	1	210		100402866								
GRIN2B/WBP11	2904/51729	7	804466	105858372									
LILRA4/LILRB1	23547/10859	8	277761		100410192								
PLVAP/COLGALT1	83483/79709	8	178245	105866266									
HERC2/CHRNA7	8924/1139	27	3708289		100401878								
SORT1/LRIG2	6272/9860	58	3675225						107971335				
ADORA2B/SPECC1	136/92521	65	4033405	105856307									
FOLH1B/HTR3A	219595/3359	134	24276357	105865103									
PMS2/GALNT17	5395/64409	285	65123299						745916				
RPL29/CLRN1	6159/7401	448	98930221		100402074								
PMS2/CUX1	5395/1523	518	95806798						100615105				

Table S2. Continued

Human genes' names	Human genes' ID	Distance (gene number)	Distance (bp)	Primate gene IDs						Evidence for neighbor fusion	Evidence for non-neighbor fusion	Cancer-associated fusions	Supporting databases and references
				M. Murinus	*C. jacchus*	*A. nancymaae*	*M. nemestrina*	*G. gorilla*	*P. troglodytes*				
PMS2/LOC100289561	5395/100289561	521	96354713						750858				
FOLH1/TRIM77	2346/390231	522	40501629					101147888					
BTF3/ACOD1	689/730249	D	D						452753				
SLC31A1/GK5	1317/256356	D	D			105723692							
PMS2/CFL1	5395/1072	D	D		100401940								
RPL32/PYCARD	6161/29108	D	D		100395878								
TAF9/NAP1L1	6880/4673	D	D		100387567								
PICALM/VBP1	8301/7411	D	D	105867622									
MTMR4/MSI1	9110/4440	D	D			105710931							
ZMYM5/CISD2	9205/493856	D	D		100409372								
NPM2/NASP	10361/4678	D	D			105716829							
MYBBP1A/CCDC197	10514/256369	D	D			105717811							
PRDX3/RPL7A	10935/6130	D	D			105714180							
SIN3A/ENOX2	25942/10495	D	D			105721607							
PPP2R3B/NPIPB2	28227/729978	D	D						112205888				
DEXI/PDCD6IP	28955/10015	D	D					101149946					
WBP11/GADL1	51729/339896	D	D	105883511									
SLC30A6/CFL1	55676/1072	D	D		100406881								
MYH7B/TUBG2	57644/27175	D	D	105877255									
SRR/HNRNPA1	63826/3178	D	D						747543				
ZSCAN16/ATMIN	80345/23300	D	D	105870590									
HM13/MCTS1	81502/28985	D	D				105496157						
TRIM5/PPIA (cypA)	85363/5478	D	D			105720336							
CCDC32/MRPL42	90416/28977	D	D	105872447									
SOWAHA/SURF4	134548/6836	D	D		100391669								
NRG4/RWDD1	145957/51389	D	D				105491035						
DENND5B/RPS27A	160518/6233	D	D		100395964								
SSBP4/ADGRA3	170463/166647	D	D					101131681					
TRIM39-RPP21/BTNL10	202658/100129094	D	D	105878814									
ADAM32/TMEM45A	203102/55076	D	D	105859347									
CPNE1/RBM12	8904/10137	I	I	105865887		105719177	105496276	101127756	100608370				
MACF1/KIAA0754	23499/643314	I	I	105879542				101146907					
MBTPS2/YY2	51360/404281	I	I					101135564					
LPCAT2/CAPNS2	54947/84290	I	I						454095				
PCDHGA3/PCDHGB1	56112/56104	I	I				105466983						
CADPS2/RNF148	93664/378925	I	I						472494				
PPP4R2/EBLN2	151987/55096	I	I				105479590						
RNLS/LIPJ	55328/142910	O	O	105875646									
DDI2/RSC1A1	84301/6248	O	O	105870486	100397033	105708556	105473217		739922				
OPN4/LDB3	94233/11155	O	O					101134517					
RNASE11/RNASE12	122651/493901	O	O				105496542						

Table S2. Continued

For each fused gene shown are the human gene names and NCBI ID's, and the number of protein-coding genes and base pairs between them. For genome assembly version from which the IDs were taken see Materials and Methods section. Zero genes (0) between Gene-1 and Gene-2 points to neighboring genes, "O" describes a known overlap between the sequences of the two neighboring genes, "I" stands for included genes (a gene-within-gene) and "D" describes two genes found on different chromosomes. Check marks and their matching references (or databases) point to existing reports on naturally occurring fusions between these genes in humans.

Reference list for Table S2:

1. Akiva, P., et al. (2006). Transcription-mediated gene fusion in the human genome. *Genome Res* 16(1): 30-36.
2. Babiceanu, M., et al. (2016). Recurrent chimeric fusion RNAs in non-cancer tissues and cells. *Nucleic Acids Res* 44(6): 2859-2872.
3. Babushok, D. V., et al. (2007). A novel testis ubiquitin-binding protein gene arose by exon shuffling in hominoids. *Genome Res* 17(8): 1129-1138.
4. Bethesda (MD): National Library of Medicine (US), National Center of Bioinformatics. NCBI Gene; Acessed on 09/2021.
5. Chen, S., et al. (2019). Widespread and Functional RNA Circularization in Localized Prostate Cancer. *Cell* 176(4): 831-843 e822.
6. Dalziel, M., et al. (2011). Alpha-MSH regulates intergenic splicing of MC1R and TUBB3 in human melanocytes. *Nucleic Acids Res* 39(6): 2378-2392.
7. Fagerberg, L., et al. (2014). Analysis of the human tissue-specific expression by genome-wide integration of transcriptomics and antibody-based proteomics. *Mol Cell Proteomics* 13(2): 397-406.
8. Flegel, C., et al. (2013). Expression profile of ectopic olfactory receptors determined by deep sequencing. *PLoS One* 8(2): e55368.
9. Huang, T. Y., et al. (2013). A novel Rac1 GAP splice variant relays poly-Ub accumulation signals to mediate Rac1 inactivation. *Mol Biol Cell* 24(3): 194-209.
10. Huret, J. L., et al. (2013). Atlas of genetics and cytogenetics in oncology and haematology in 2013. *Nucleic Acids Res* 41(Database issue): D920-924.
11. Kim, P. and X. Zhou (2019). FusionGDB: fusion gene annotation DataBase. *Nucleic Acids Res* 47(D1): D994-D1004.
12. Kinsella, M., et al. (2011). Sensitive gene fusion detection using ambiguously mapping RNA-Seq read pairs. *Bioinformatics* 27(8): 1068-1075.
13. Klijn, C., et al. (2015). A comprehensive transcriptional portrait of human cancer cell lines. *Nat Biotechnol* 33(3): 306-312.
14. Lopez-Nieva, P., et al. (2019). Detection of novel fusion-transcripts by RNA-Seq in T-cell lymphoblastic lymphoma. *Sci Rep* 9(1): 5179.
15. Nacu, S., et al. (2011). Deep RNA sequencing analysis of readthrough gene fusions in human prostate adenocarcinoma and reference samples. *BMC Med Genomics* 4: 11.
16. Ou, M. Y., et al. (2021). The CTNNBIP1-CLSTN1 fusion transcript regulates human neocortical development. *Cell Rep* 35(13): 109290.
17. Parra, G., et al. (2006). Tandem chimerism as a means to increase protein complexity in the human genome. *Genome Res* 16(1): 37-44.
18. Pintarelli, G., et al. (2016). Read-through transcripts in normal human lung parenchyma are down-regulated in lung adenocarcinoma. *Oncotarget* 7(19): 27889-27898.
19. Prakash, T., et al. (2010). Expression of conjoined genes: another mechanism for gene regulation in eukaryotes. *PLoS One* 5(10): e13284.
20. Stephens, P. J., et al. (2009). Complex landscapes of somatic rearrangement in human breast cancer genomes. *Nature* 462(7276): 1005-1010.
21. Tate, J. G., et al. (2019). COSMIC: the Catalogue Of Somatic Mutations In Cancer. *Nucleic Acids Res* 47(D1): D941-D947.
22. Wang, J., et al. (2019). Interfering Expression of Chimeric Transcript SEPT7P2-PSPH Promotes Cell Proliferation in Patients with Nasopharyngeal Carcinoma. *J Oncol* 2019: 1654724.
23. Wen, H., et al. (2012). New fusion transcripts identified in normal karyotype acute myeloid leukemia. *PLoS One* 7(12): e51203.

Table S3. Number of donor samples contributing to gene expression data in each tissue listed in the GTEx database V7.

Tissue sample description	Number of donors	Tissue sample description	Number of donors
Adipose: Subcutaneous	442	Esophagus: Gastroesophageal	244
Adipose: Visceral (Omentum)	355	Esophagus: Mucosa	407
Adrenal Gland	190	Esophagus: Muscularis	370
Artery: Aorta	299	Fallopian Tube*	7
Artery: Coronary	173	Heart: Atrial Appendage	297
Artery: Tibial	441	Heart: Left Ventricle	303
Bladder	11	Kidney Cortex	45
Brain: Amygdala	100	Liver	175
Brain: Anterior cingulate cortex BA24	121	Lung	427
Brain: Caudate basal ganglia	160	Minor Salivary Gland	97
Brain: Cerebellar Hemisphere	136	Muscle Skeletal	564
Brain: Cerebellum	173	Nerve Tibial	414
Brain: Cortex	158	Ovary[#]	133
Brain: Frontal Cortex BA9	129	Pancreas	248
Brain: Hippocampus	123	Pituitary	183
Brain: Hypothalamus	121	Prostate	152
Brain: Nucleus accumbens basal ganglia	147	Skin: Not Sun Exposed Suprapubic	387
Brain: Putamen basal ganglia	124	Skin: Sun Exposed Lower leg	473
Brain: Spinal cord cervical	91	Small Intestine: Terminal Ileum	137
Brain: Substantia nigra	88	Spleen	162
Breast Mammary Tissue	290	Stomach	262
Cells: lymphocytes	130	Testis[#]	259
Cells: Transformed fibroblasts	343	Thyroid	446
Cervix: EctoCervix*	6	Uterus	111
Cervix: EndoCervix*	5	Vagina	115
Colon: Sigmoid	233	Whole Blood	407
Colon: Transverse	274		

*) Tissues removed from the analyses, since their expression data came from less than ten donors
#) Germline tissues removed from the soma co-expression analysis
